# Supplementary material for: Temporal variation in the diagnosis of resolved atrial fibrillation and the influence of performance targets on clinical coding: cohort study
Source: BMJ Open. 2019 Nov 21;9(11):e030454. doi: 10.1136/bmjopen-2019-030454 (PMC6887086; doi:10.1136/bmjopen-2019-030454)
Supplement: Author's manuscript [file bmjopen-2019-030454.draft_revisions.pdf]

BMJ Open

BMJ Open is committed to open peer review. As part of this commitment we make the peer review history of every article we publish publicly available.

When an article is published we post the peer reviewers' comments and the authors' responses online. We also post the versions of the paper that were used during peer review. These are the versions that the peer review comments apply to.

The versions of the paper that follow are the versions that were submitted during the peer review process. They are not the versions of record or the final published versions. They should not be cited or distributed as the published version of this manuscript.

BMJ Open is an open access journal and the full, final, typeset and author-corrected version of record of the manuscript is available on our site with no access controls, subscription charges or pay-per-view fees (http://bmjopen.bmj.com).

If you have any questions on BMJ Open's open peer review process please email info.bmjopen@bmj.com

BMJ Open

Temporal variation in the diagnosis of resolved atrial fibrillation and the influence of performance targets on clinical coding: cohort study

Journal:	*BMJ Open*
Manuscript ID	bmjopen-2019-030454
Article Type:	Research
Date Submitted by the Author:	15-Mar-2019
Complete List of Authors:	Adderley, Nicola; University of Birmingham, Institute of Applied Health Research<br>Nirantharakumar, Krishnarajah; University of Birmingham, Institute of Applied Health Research<br>Marshall, Tom; University of Birmingham, Institute of Applied Health Research
Keywords:	PRIMARY CARE, CARDIOLOGY, Anticoagulation < HAEMATOLOGY

SCHOLARONE™
Manuscripts

TITLE

Temporal variation in the diagnosis of resolved atrial fibrillation and the influence of performance targets on clinical coding: cohort study

AUTHORS

Nicola J Adderley[1], Krishnarajah Nirantharakumar[2], Tom Marshall[3]

[1]Research Fellow, Institute of Applied Health Research, University of Birmingham, Edgbaston, Birmingham, B15 2TT

[2]Senior Clinical Lecturer, Institute of Applied Health Research, University of Birmingham, Edgbaston, Birmingham, B15 2TT

[3]Professor of Public Health and Primary Care, Institute of Applied Health Research, University of Birmingham, Edgbaston, Birmingham, B15 2TT

Corresponding author

Tom Marshall

Address: Institute of Applied Health Research, University of Birmingham, Edgbaston, Birmingham, B15 2TT

Email: t.p.marshall@bham.ac.uk

WORD COUNT

Word count (abstract):	276
Word count (main text):	3351
Tables:	0
Figures:	3
References:	15

For peer review only

For peer review only - http://bmjopen.bmj.com/site/about/guidelines.xhtml

ABSTRACT

Objectives

To investigate how the introduction of performance targets for anticoagulation in atrial fibrillation (AF) affected use of the 'resolved atrial fibrillation' code.

Design

Retrospective cohort studies.

Setting

Data from The Health Improvement Network (THIN), a UK database of electronic patient records, from 2000 to 2016.

Participants

250,788 adult patients aged ≥18 years with a diagnosis of AF, including 14,757 with an incident diagnosis of 'resolved AF'.

Main outcome measures

Annual and monthly incidence of 'resolved AF' from 2000 to 2016. Among patients with 'resolved AF', for each year we calculated median duration of the preceding AF diagnosis and the proportion prescribed anticoagulants prior to 'resolved AF'.

Results

Incidence of 'resolved AF' increased from 5.7 to 26.3 per 1000 person-years between 2005 and the introduction of AF performance targets in 2006. Since 2007, monthly incidence has been highest between January and March. Among 'resolved AF' patients with CHA_2DS_2-VASc score ≥1, 81.9% (95%CI 81.1 to 82.6) had no current anticoagulant prescription, and 62.3% (95%CI 61.4 to 63.2) had no record of any anticoagulant prescription.

Conclusion

The introduction of AF performance targets was followed by a large increase in use of the 'resolved AF' code; use of the code is highest in the months immediately before practices make their anticoagulant performance target submissions. Although most AF patients are prescribed anticoagulants, few patients diagnosed with 'resolved AF' are prescribed anticoagulants and most have never been prescribed them. Untreated patients are much more likely to be coded as having 'resolved AF'. This suggests general practices are choosing to code some patients as having 'resolved AF', thereby removing these patients from the AF register, in order to improve their apparent performance.

For peer review only

For peer review only - http://bmjopen.bmj.com/site/about/guidelines.xhtml

Strengths and limitations of this study

- Analysis was performed in a large primary care dataset which is generalisable to the UK population and included more than a quarter of a million patients with atrial fibrillation (AF).
- Data was derived from routinely clinical data which is used by general practitioners for clinical decision-making.
- The study assessed the impact of the introduction of AF into the Quality and Outcomes Framework on the use of the 'resolved AF' clinical code.
- Use and interpretation of the 'resolved AF' code is likely to vary between general practitioners and practices.
- The primary care dataset contains no direct information on general practitioners' reasons for assigning a 'resolved AF' code; possible influencing factors have therefore been inferred from explorations of temporal variation, patient diagnostic information and anticoagulant prescribing.

For peer review only

For peer review only - http://bmjopen.bmj.com/site/about/guidelines.xhtml

INTRODUCTION

Atrial fibrillation (AF) is a common cardiac arrhythmia associated with increased risk of stroke and transient ischaemic attack (TIA); this increased risk is attenuated by treatment with anticoagulants.[1,2,3] AF may be categorised as resolved if normal heart rhythm is restored. However, AF may recur after apparent resolution. [4,5] Evidence shows that patients diagnosed as having 'resolved AF' continue to be at increased risk of stroke/TIA; from 2013 to 2016, risk in patients with 'resolved AF' was found to be the same as that in patients with ongoing AF.[6]

Factors influencing clinicians to make a diagnosis of 'resolved AF' are unclear. Research has demonstrated that the prevalence of the AF diagnosed clinical code in UK general practice increased significantly after 2006 and has remained comparatively high since.[6] AF was introduced into the Quality and Outcomes Framework (QOF) in 2006,[7] suggesting that QOF may have contributed to the increase in prevalence of 'resolved AF' diagnoses. There was no corresponding jump in the recorded prevalence of AF at this time.[8] From April 2006, general practices were required to maintain a register of patients with AF and to record whether eligible patients were prescribed anticoagulants or antiplatelets. In 2012, the AF QOF indicators were updated to include an assessment of stroke risk and to require patients with a high stroke risk to be treated with anticoagulants (not antiplatelets).[9]

We hypothesised that the introduction of AF into QOF had an impact on the use of the 'resolved AF' code. The aim of this analysis, therefore, was to use information available in routinely collected primary care data to explore this hypothesis by investigating variation in the use of the 'resolved AF' clinical code over time and across different practices, and to investigate other factors which may influence general practitioners to assign a diagnosis of 'resolved AF'. The specific questions addressed were:

1. What is the annual incidence of 'resolved AF' diagnoses and did incidence increase with the introduction of AF into QOF?
2. Since the introduction of AF into QOF, is a diagnosis of 'resolved AF' more likely to be recorded in the months of January to March, immediately prior to the practice QOF submission?
3. Is there a difference in the duration of AF diagnosis in patients diagnosed as having 'resolved AF' before and after the introduction of AF into QOF?
4. Are patients prescribed anticoagulants before their 'resolved AF' diagnosis?
5. How much variation exists between general practices in use of the 'resolved AF' code?

Evidence indicating that use of the 'resolved AF' code is substantially driven by QOF reporting would support the recommendation that patients with 'resolved AF' be included in QOF AF registers and receive ongoing AF management,[6] or that the 'resolved AF' clinical code be withdrawn.

METHODS

Data source

Datasets were extracted from The Health Improvement Network (THIN), a database of electronic primary care records from UK general practices using Vision software. The version of the database from which study datasets were derived included data for approximately 14 million patients at over

For peer review only - http://bmjopen.bmj.com/site/about/guidelines.xhtml

640 practices. THIN comprises coded data on patient demographics, diagnoses, prescriptions issued in primary care, consultations and investigations.

Population

General practices were eligible for participation from the later of the practice acceptable mortality recording (AMR) date,[10] Vision installation date plus one year, and the study start date (1 year prior to the first index/census date).

All adult patients aged 18 years and over with a recorded diagnosis of atrial fibrillation and registered for at least 365 days before the index/census date were eligible for inclusion. AF was defined by a record of a relevant clinical (Read) code.

Study design

A retrospective cohort study from 1st January 2000 to 31st December 2016 was carried out. Index date was the latest of the following two dates: one year after the patient registered with the practice or the date of diagnosis of AF.

To determine incidence of 'resolved AF' among patients with AF, eligible patients were followed up from the index date until the earliest of the following: patient left the practice/transferred out, death, study end date, most recent data upload from practice, or a diagnosis of 'resolved AF'. Patients with a record of 'resolved AF' at study entry were excluded. 'Resolved AF' was defined as a record of the relevant clinical (Read) code (212R.00 'Atrial fibrillation resolved').[6]

To explore temporal variation in AF duration and anticoagulant prescribing preceding a diagnosis of 'resolved AF', a cohort restricted to patients with a diagnosis of 'resolved AF' during the study period was used. Eligible patients were followed up until the earliest of the following: patient left practice/transferred out, death, study end date, most recent data upload from practice, or an outcome event.

To explore practice-level variation in use of the 'resolved AF' clinical code, a cross-sectional study was carried out on 1st December 2016.

Analysis

Annual incidence of 'resolved AF'

Annual incidence rates of a 'resolved AF' diagnosis among AF patients were calculated for each year from 2000 to 2016 by dividing the number of patients with a new (first) record of 'resolved AF' (numerator) by the total number of person-years at risk (denominator) for the given year.

Monthly variation in use of the 'resolved AF' code pre- and post-QOF

To investigate the impact of QOF on the distribution of 'resolved AF' coding throughout the year, monthly incidence of 'resolved AF' diagnoses (in each month from January to December) was calculated in the pre-QOF period (2000 to 2005), in 2006 and 2007, and in the post-QOF period

For peer review only - http://bmjopen.bmj.com/site/about/guidelines.xhtml

(2008 to 2016). Monthly incidence was calculated separately for 2006 and 2007 as annual incidence of 'resolved AF' in this period, the years of and immediately following the introduction of AF into QOF, was found to be substantially higher than in subsequent years.

In the post-QOF period (2007 onwards), Poisson regression was used to calculate crude and adjusted incidence rate ratios of stroke/transient ischaemic attack (TIA) in patients with a 'resolved AF' diagnosis recorded in January to March compared to April to December, in order to explore any possible differences in disease severity between patients coded as resolved at different times of the year. The adjusted model included the following covariates: age, sex, CHA_2DS_2-VASc score (categorised as 0, 1 ≥2) and prescription of anticoagulant medication at the time of the 'resolved AF' diagnosis.

'Resolved AF' cohort

The following analyses were restricted to patients with a record of 'resolved AF'.

Duration of AF diagnosis

To explore variation over time in duration of AF diagnosis in patients with 'resolved AF', median (interquartile range, IQR) duration of time between diagnosis of AF (earliest recorded Read code) and first record of a 'resolved AF' code was calculated for each year in patients with a 'resolved AF' code.

Anticoagulant prescribing

To explore prescribing of anticoagulants to patients with a diagnosis of 'resolved AF', the proportion of patients on anticoagulant treatment at the time of diagnosis (current treatment, prescribed up to 90 days prior to 'resolved AF' record), 0 to 90 days, and 91 to 180 days after the 'resolved AF' diagnosis were calculated with 95% CIs for proportions in 1) all 'resolved AF' patients and 2) patients with a CHA_2DS_2-VASc score ≥1 (eligible for anticoagulant treatment). The proportion of 'resolved AF' patients with a CHA_2DS_2-VASc score ≥1 who had never been prescribed anticoagulants was also calculated. Trends over time were explored by calculating the proportions for each year between 2000 and 2016.

Cross-sectional analysis

Practice-level variation in use of 'resolved AF' code

Variation in use of the 'resolved AF' code by general practice in 2016 was assessed by plotting the percentage of AF patients with any record of a 'resolved AF' code (ever) at a given practice against the number of AF patients at the practice. Upper and lower control limits (within 3 standard deviations of the mean) were calculated.

For peer review only - http://bmjopen.bmj.com/site/about/guidelines.xhtml

For peer review only

Definitions of variables

AF, 'resolved AF', and stroke/TIA were defined by the presence of a clinical code; the absence of a clinical code was taken to indicate no diagnosis. The clinical code lists used have been utilised in a number of previous AF studies[6,8,11,12,13] and include all codes used in QOF.[14]

CHA_2DS_2-VASc scores were calculated by adding 1 point each for a history of congestive heart failure (HF), hypertension, diabetes (DM), vascular disease, age 65-74 years and female sex (if another risk factor was present, otherwise 0), and 2 points for age ≥75 and a history of stroke/TIA. HF, hypertension, DM and vascular disease were defined by a relevant clinical code.

Anticoagulants included warfarin, parenteral anticoagulants, other vitamin K antagonists, and novel/non-vitamin K oral anticoagulants.

All statistical analyses were performed in Stata IC version 14.2.

Patient involvement

Patients were not involved in the research.

RESULTS

Annual incidence of 'resolved AF'

A total of 250,788 patients with AF contributing 1,037,858 person-years were included in the analysis; 14,757 patients had an incident diagnosis of 'resolved AF'. Mean (SD) age was 74.6 (12.1) years; 52.6% pf patients were male; median (IQR) follow-up was 3.1 (1.2-6.1) years.

Incidence of the atrial fibrillation (AF) resolved code in patients with AF showed a sharp rise in 2006 (Figure 1), at which time AF was introduced into QOF, rising from 5.7 per 1000 person-years in 2005 to 26.3 per 1000 person-years in 2006. Incidence peaked at 28.6 per 1000 person-years in 2007; it declined thereafter, before rising again to 19.5 per 1000 person-years in 2012-13, when further changes were made to the QOF AF requirements. Since 2013 the incidence has declined.

Monthly variation in use of the 'resolved AF' code

Prior to the introduction of AF into QOF (January 2000 to March 2006), incidence of the 'resolved AF' code remained relatively constant across the 12 months of the year, including the 3 months immediately prior to the introduction of AF into QOF (January to March 2006), with monthly incidence varying between 3.2 and 7.2 per 1000 person-years (Figure 2). From April 2006 and for the subsequent 12 months, incidence of the code steadily increased, reaching a peak of 70.2 per 1000 person-years in January 2007. From 2007 onwards (post-QOF), incidence of the 'resolved AF' code has been highest between the months of January and March, the 3 months immediately preceding QOF report submission. In the post-QOF period (2008 to 2016) incidence is higher in every month of the year relative to the same month in the pre-QOF period.

For peer review only - http://bmjopen.bmj.com/site/about/guidelines.xhtml

From 2007 onwards, 245 patients diagnosed with 'resolved AF' in January to March and 358 patients diagnosed in April to December had a stroke. Crude incidence rates were 12.4 and 13.8 per 1000 person-years, respectively. Among patients who received a diagnosis of 'resolved AF' after the introduction of AF into QOF (2007 onwards), there was no difference in incidence of stroke/TIA in patients who were assigned the code between January and March compared to those given the code later in the year: crude IRR 0.90 (95% CI 0.76 to 1.06), adjusted IRR 0.98 (95% CI 0.83 to 1.15).

'Resolved AF' cohort

14,863 patients with a record of 'resolved AF' were included in the cohort from 2000 to 2016. Median (IQR) age was 70.7 (59.6-79.6); 58.1% of patients were male. 11,479 (77.2%) patients had a CHA_2DS_2-VASc score ≥1.

Duration of time between diagnosis of AF and use of the 'resolved AF' code

Median duration of time between diagnosis of AF and first recording of a 'resolved AF' code remained between several months and approximately a year (varying from 69 to 335 days) between 2000 and 2005. In 2006 there was a sharp rise in median duration from 276 days (9 months) in 2005 to 1343 days (3 years 8 months) in 2006. This indicates that in 2006 more than half of patients who were assigned a 'resolved AF' code had been diagnosed over 3 ½ years earlier. Median duration then declined for several years, before rising again to more than 1000 days in 2012-13.

Sequence of events in relation to anticoagulant prescribing in 'resolved AF' patients

Few patients were still on anticoagulants when the 'resolved AF' code was recorded. In the cohort of 'resolved AF' patients (2000 to 2016), 17.3% (95% CI 16.7 to 17.9) had a current prescription at the time of 'resolved AF' recording (up to 90 days prior), with 82.7% (95% CI 82.1 to 83.3) not being prescribed anticoagulant treatment. Up to 90 days following the 'resolved AF' diagnosis, 9.8% (95% CI 9.3 to 10.3) of patients were still being prescribed anticoagulants. By 91 to 180 days after 'resolved AF', 8.7% (95% CI 8.3 to 9.2) had a prescription for anticoagulants.

Among 'resolved AF' patients with a CHA_2DS_2-VASc score ≥1, 18.1% (95% CI 17.4 to 18.9) had a current prescription for anticoagulants, while 81.9% (95% CI 81.1 to 82.6) had no current prescription. 10.5% (95% CI 10.0 to 11.1) and 9.7% (95% CI 9.2 to 10.3) had prescriptions up to 90 days and 91 to 180 days following the 'resolved AF' diagnosis respectively. The proportion of 'resolved AF' patients prescribed anticoagulants shortly before and after recording of the 'resolved AF' code varied slightly over time, with a notable drop in 2006 to 9.8% (95% CI 8.5 to 11.4), decreasing from 25.2% (95% CI 20.6 to 30.3) in 2005.

62.3% (95% CI 61.4 to 63.2) of 'resolved AF' patients with a CHA_2DS_2-VASc score ≥1 had no record of an anticoagulant prescription. Among the cohort of patients whose first record of AF was after registration with the practice (n=13,307), 60.6% (95% CI 59.6 to 61.5) had never been prescribed anticoagulants; this proportion varied slightly over time, reaching a peak of 70.2% in 2006 and a low of 51.3% in 2016.

For peer review only - http://bmjopen.bmj.com/site/about/guidelines.xhtml

Practice-level variation

787 practices with a total of 1,167,771 patients with AF were included in the analysis from 2000 to 2016. 443 practices with a total of 69,262 patients with AF, of whom 7,261 had a record of 'resolved AF', were included in the analysis in 2016.

Variation in use of the 'resolved AF' code between general practices

The proportion of AF patients with a record of 'resolved AF' varied between practices, ranging from 0% to 43% in 2016. The majority of practices fell within the acceptable range (between the upper (UCL) and lower (LCL) control limits) based on the size of the practice AF population, although a number of practices fell outside this range: 54 (12.2%) practices above the UCL and 30 (6.8%) below the LCL (Figure 3). In 2016, 3 practices with more than 100 patients with AF assigned a 'resolved AF' code to none of these patients, while 10 practices assigned a 'resolved AF' code to more than 25% of patients with AF.

Similar patterns in variation were observed in the year immediately after the introduction of AF into QOF (2007): the proportion of patients with 'resolved AF' ranged from 0% to 40%, with 61 (13.8%) practices above the UCL and 30 (6.8%) below the LCL. In 2005, immediately before the introduction of AF into QOF, there was slightly less variation: the proportion of patients with 'resolved AF' ranged from 0% to 30%, with 39 (8.8%) practices above the UCL. None were below the LCL, which was low due to the smaller average number of patients with 'resolved AF'.

DISCUSSION

Incidence of 'resolved AF' rose dramatically in 2006 immediately following the introduction of AF into the Quality and Outcomes Framework (QOF).[7] Incidence peaked the following year at 28.6 per 1000 person-years, showing a five-fold increase compared to the incidence prior to QOF. There was a further, smaller, peak in 'resolved AF' incidence in 2012-13, following a change in the QOF AF indicators to introduce a stroke risk assessment indicator and to change the requirements for the anticoagulation indicator.[9] A corresponding rise in the prevalence of 'resolved AF' among patients with AF, from 2.3% in 2005 to 6.4% in 2007 and a high of 9.2% in 2013, has been reported previously.[6]

Since the introduction of AF into QOF, the majority of 'resolved AF' codes have been recorded between the months of January and March, immediately prior to QOF report submission by general practices. Prior to this, 'resolved AF' codes were recorded throughout the year with little monthly variation in incidence. There is no difference in stroke/TIA rates in patients diagnosed as having 'resolved AF' between January and March compared to those diagnosed later in the year; patients with AF who are diagnosed as resolved immediately prior to QOF do not have a different/lower risk of stroke/TIA.

Immediately following the introduction of AF into QOF, there was a dramatic rise in median duration between AF and 'resolved AF' diagnoses, with a further peak at the time of changes to QOF in 2012-13. At these time points, patients designated as having 'resolved AF' had been diagnosed with AF

For peer review only - http://bmjopen.bmj.com/site/about/guidelines.xhtml

several years previously (median 3 years and 8 months in 2006) compared around one year prior to QOF (9 months in 2005).

Almost two thirds of patients with ‘resolved AF’ and a CHA_2DS_2-VASc score ≥1 had never been prescribed anticoagulants. In 2016, 79.5% of patients with ‘resolved AF’ and a CHA_2DS_2-VASc score ≥1 were not prescribed anticoagulants at the time of their ‘resolved AF’ diagnosis, made up of 53.5% who had never been prescribed anticoagulants and 26.0% who had previously been prescribed anticoagulants but had subsequently discontinued. By contrast, only 25-30% of patients with ongoing AF and a CHA_2DS_2-VASc score ≥1 were not prescribed anticoagulants in 2016.[8,15] This suggests that patients with AF who are not prescribed anticoagulants may be more likely to be assigned a ‘resolved AF’ code. Furthermore, recent evidence indicates that patients with a diagnosis of ‘resolved AF’ remain at increased risk of stroke/TIA and may therefore benefit from continued anticoagulant prophylaxis.[6]

Use of the ‘resolved AF’ code varies between practices. Some practices with large numbers of AF patients use the code for very few patients, while others assign the code to more than a quarter of AF patients.

Strengths and limitations

This analysis was performed in a large general practice dataset which is generalisable to the UK population. Data was derived from routinely clinical data which is used by general practitioners for clinical decision-making. The use and interpretation of the ‘resolved AF’ clinical code is likely to vary between general practitioners and practices. The primary care dataset contains no direct information on general practitioners’ reasons for assigning a ‘resolved AF’ code; possible influencing factors have therefore been inferred from explorations of temporal variation, patient diagnostic information and anticoagulant prescribing. In order to better understand the factors motivating a diagnosis of ‘resolved AF’, a qualitative study and consultation with practicing clinicians would be required.

Conclusions

This research highlights that the introduction of AF into the Quality and Outcomes Framework has substantially influenced general practitioners’ decisions with regard to assigning a ‘resolved AF’ clinical code. Incidence of the ‘resolved AF’ code increased significantly in 2006 to 2007, at the time AF was introduced into QOF; incidence increased again, to a lesser extent, between 2012 and 2013, when further changes were made to the QOF indicators. Since AF was introduced into QOF, incidence of the ‘resolved AF’ code is highest in the months shortly before practices make their QOF submissions. Use of the code remains common.

60% of patients who are assigned a ‘resolved AF’ code and who have a CHA_2DS_2-VASc score ≥1 have never been prescribed anticoagulants. Very few patients (17.3%) are still taking anticoagulants at the time the ‘resolved AF’ code is recorded, suggesting that patients with AF who are not being prescribed anticoagulants may be more likely to be diagnosed as ‘resolved’.

This evidence suggests that in order to improve their apparent performance general practices are choosing to code some patients with atrial fibrillation as being ‘resolved’, particularly those not receiving anticoagulants, thereby removing these patients from the AF register. Previous evidence has demonstrated that patients with a diagnosis of ‘resolved AF’ remain at increased risk of

For peer review only - http://bmjopen.bmj.com/site/about/guidelines.xhtml

stroke/TIA and are likely to benefit from anticoagulant prophylaxis. We therefore recommend that the 'resolved AF' clinical code be withdrawn as an exclusion criterion from the Quality and Outcomes Framework, or that performance indicators for anticoagulant prophylaxis in AF be extended to patients diagnosed as having 'resolved AF'.

For peer review only

For peer review only - http://bmjopen.bmj.com/site/about/guidelines.xhtml

FIGURE LEGENDS

Figure 1. Annual incidence of resolved atrial fibrillation in patients with AF 2000-2016.

Figure 2. Incidence of the 'resolved AF' code by month of recording, before, during and after the introduction of AF into the Quality and Outcomes Framework (QOF).

Figure 3. Funnel plot showing variation in use of the 'resolved AF' code by practice in 2016.

ETHICAL APPROVAL

The THIN data collection scheme and research carried out using THIN data were approved by the NHS South-East Multicentre Research Ethics Committee (MREC) in 2003; under the terms of this approval, studies must undergo independent scientific review. Approval for these analyses was obtained from the Scientific Review Committee (for the use of THIN data) in April 2015 (15THIN021) and September 2017 (SRC reference number 17THIN082).

COMPETING INTERESTS

All authors have completed the ICMJE uniform disclosure form at http://www.icmje.org/coi_disclosure.pdf and declare: NA and TM report a grant from the National Institute for Health Research (NIHR) Collaboration for Leadership in Applied Health Research and Care West Midlands during the conduct of the study; KN reports funding from AstraZeneca and fees from Sanofi and Boehringer Ingelheim outside the submitted work. Authors declare no other financial relationships with any organisations that might have an interest in the submitted work in the previous three years; and no other relationships or activities that could appear to have influenced the submitted work.

CONTRIBUTORS

NA, KN and TM designed the study. KN undertook data extraction. NA designed and performed the analyses. NA wrote the first draft of the paper, which was revised in collaboration with TM and KN. NA acts as guarantor.

FUNDING

NJA and TM were funded by the NIHR Collaboration for Leadership in Applied Health Research and Care West Midlands initiative (NIHR CLAHRC-WM). This paper presents independent research and the views expressed are those of the author(s) and not necessarily those of the NHS, the NIHR or the Department of Health.

The funders had no role in study design, data collection and analysis, decision to publish, or preparation of the manuscript.

For peer review only

TRANSPARENCY

The lead author (the manuscript's guarantor) affirms that the manuscript is an honest, accurate, and transparent account of the study being reported; that no important aspects of the study have been omitted; and that any discrepancies from the study as planned (and, if relevant, registered) have been explained.

DATA SHARING STATEMENT

Dataset is not available.

EXCLUSIVE LICENCE

I, the Submitting Author has the right to grant and does grant on behalf of all authors of the Work (as defined in the below author licence), an exclusive licence and/or a non-exclusive licence for contributions from authors who are: i) UK Crown employees; ii) where BMJ has agreed a CC-BY licence shall apply, and/or iii) in accordance with the terms applicable for US Federal Government officers or employees acting as part of their official duties; on a worldwide, perpetual, irrevocable, royalty-free basis to BMJ Publishing Group Ltd ("BMJ") its licensees and where the relevant Journal is co-owned by BMJ to the co-owners of the Journal, to publish the Work in BMJ Open and any other BMJ products and to exploit all rights, as set out in our licence.

For peer review only

REFERENCES

[1] Antithrombotic Trialists' Collaboration. Collaborative meta-analysis of randomised trials of antiplatelet therapy for prevention of death, myocardial infarction, and stroke in high risk patients. *BMJ* 2002; 324:71–86.

[2] Aguilar MI and Hart R. Oral anticoagulants for preventing stroke in patients with non-valvular atrial fibrillation and no previous history of stroke or transient ischemic attacks. Cochrane Database of Systematic Reviews 2005, Issue 3. Art. No.: CD001927. doi: 10.1002/14651858.CD001927.pub2.

[3] Aguilar MI, Hart R and Pearce LA. Oral anticoagulants versus antiplatelet therapy for preventing stroke in patients with non-valvular atrial fibrillation and no history of stroke or transient ischemic attacks. *Cochrane Database of Systematic Reviews* 2007, Issue 3. Art. No.: CD006186. doi: 10.1002/14651858.CD006186.pub2.

[4] Wynn GJ, El-Kadri M, Haq I, Das M, Modi S, Snowdon R, Hall M, Waktare JE, Todd DM, Gupta D. Long-term outcomes after ablation of persistent atrial fibrillation: an observational study over 6 years. Open Heart. 2016;3:e000394. doi: 10.1136/openhrt-2015-000394

[5] Militaru C, Donoiu I. Atrial Fibrillation Recurrence Predictors after Conversion to Sinus Rhythm Curr Health Sci J. 2014 Apr-Jun; 40(2): 129–133. doi:10.12865/CHSJ.40.02.09

[6] Adderley NJ, NIrantharakumar K, Marshall T. Risk of stroke and transient ischaemic attack in patients with a diagnosis of resolved atrial fibrillation: retrospective cohort studies. BMJ 2018. doi:10.1136/bmj.k1717

[7] NHS Employers and General Practitioners Committee. Revisions to the GMS contract 2006/07. Delivering investment in general practice. NHS Employers: London 2006.

[8] Adderley NJ, Ryan R, Nirantharakumar K, Marshall T. Prevalence and treatment of atrial fibrillation in UK general practice from 2000 to 2016. Heart 2018. doi:10.1136/heartjnl-2018-312977

[9] NHS Employers and General Practitioners Committee. Quality and Outcomes Framework for 2012/13. Guidance for PCOs and practices. NHS Employers: London 2012.

[10] Maguire A, Blak BT, Thompson M. The importance of defining periods of complete mortality reporting for research using automated data from primary care. *Pharmacoepidemiol Drug Saf* 2009;18:76e83.

[11] Cowan C, Healicon R, Robson I, et al. The use of anticoagulants in the management of atrial fibrillation among general practices in England. *Heart* 2013;99:1166–72. doi: 10.1136/heartjnl-2012-303472

[12] Adderley N, Ryan R, Marshall T. The role of contraindications in prescribing anticoagulants to patients with atrial fibrillation: a cross-sectional analysis of primary care data in the UK. *Br J Gen Prac* 2017. doi: 10.3399/bjgp17X691685

[13] Isaew A, Adderley NJ, Ryan R, Fitzmaurice D, Marshall T. The treatment of paroxysmal atrial fibrillation in UK primary care. *Heart* 2017;103:1502-7. doi: 10.1136/heartjnl-2016-310927

[14] NHS England. New GMS Contract QOF Implementation. Dataset and Business Rules. Atrial Fibrillation Indicator Set. Leeds: Health and Social Care Information Centre 2016.

[15] Lacoin L, Lumley M, Ridha E, Pereira M, McDonald L, Ramagopalan S, et al. Evolving landscape of stroke prevention in atrial fibrillation within the UK between 2012 and 2016: a cross-sectional analysis study using CPRD. BMJ Open 2017;7:e015363. doi:10.1136/bmjopen-2016-015363

For peer review only - http://bmjopen.bmj.com/site/about/guidelines.xhtml

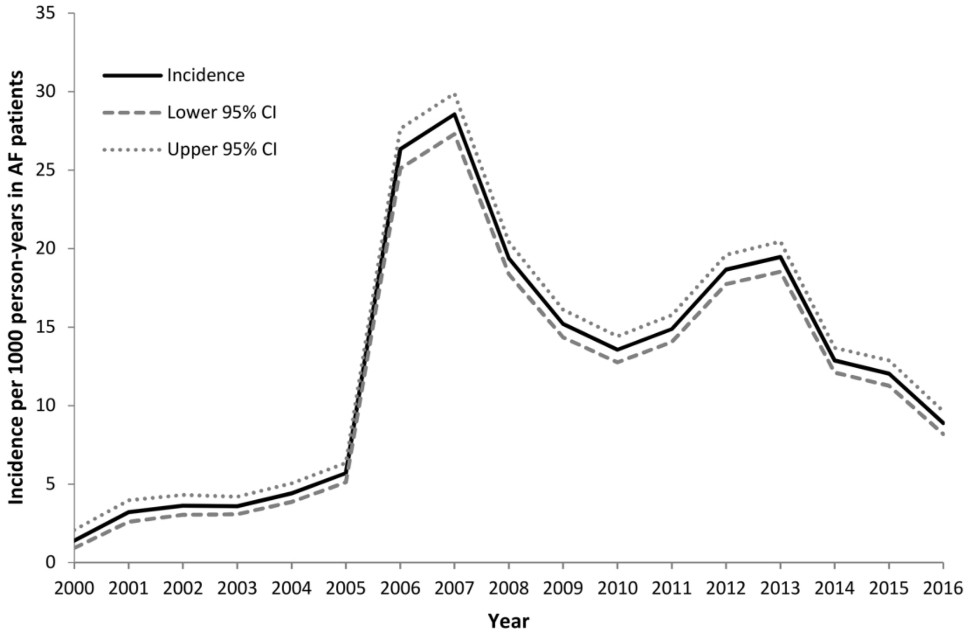

Figure 1. Annual incidence of resolved atrial fibrillation in patients with AF 2000-2016.

157x105mm (300 x 300 DPI)

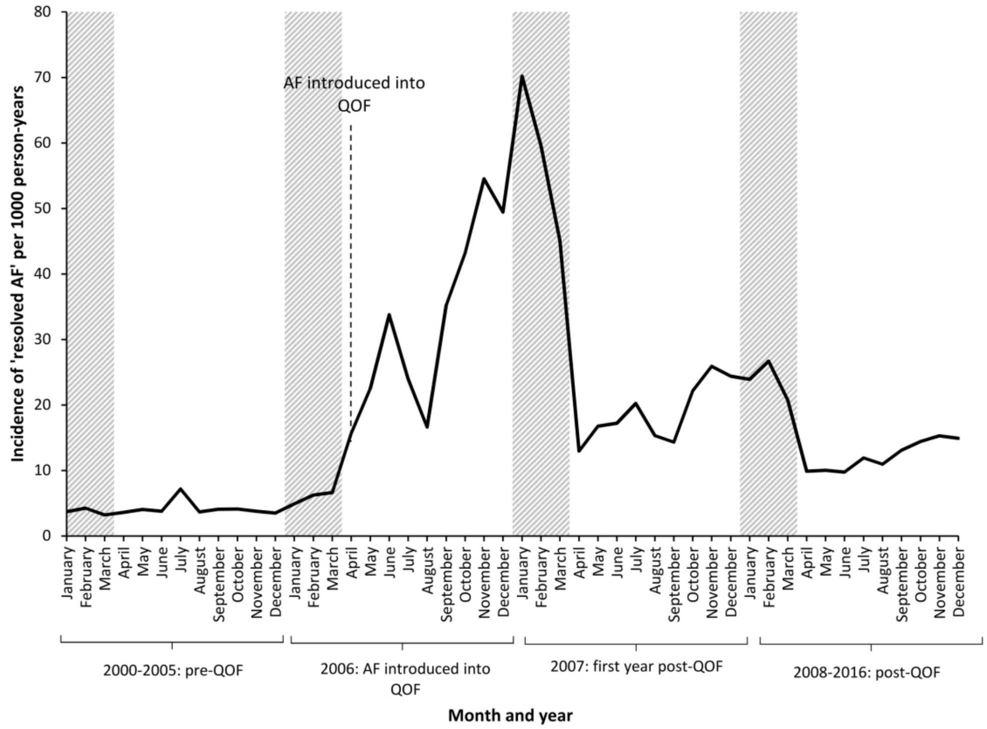

Figure 2. Incidence of the 'resolved AF' code by month of recording, before, during and after the introduction of AF into the Quality and Outcomes Framework (QOF).

188x138mm (300 x 300 DPI)

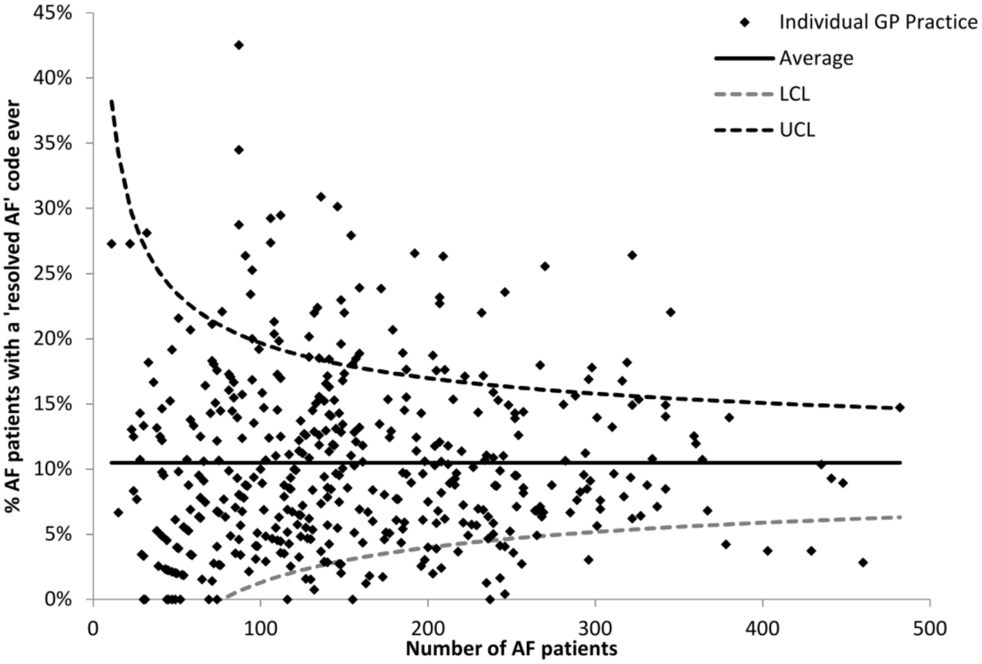

Figure 3. Funnel plot showing variation in use of the 'resolved AF' code by practice in 2016.

155x104mm (300 x 300 DPI)

The RECORD statement – checklist of items, extended from the STROBE statement, that should be reported in observational studies using routinely collected health data.

	Item No.	STROBE items	Location in manuscript where items are reported	RECORD items	Location in manuscript where items are reported
**Title and abstract**					
	1	(a) Indicate the study's design with a commonly used term in the title or the abstract (b) Provide in the abstract an informative and balanced summary of what was done and what was found	p.3	RECORD 1.1: The type of data used should be specified in the title or abstract. When possible, the name of the databases used should be included.<br><br>RECORD 1.2: If applicable, the geographic region and timeframe within which the study took place should be reported in the title or abstract.<br><br>RECORD 1.3: If linkage between databases was conducted for the study, this should be clearly stated in the title or abstract.	p.3<br><br>p.3<br><br>n/a
**Introduction**					
Background rationale	2	Explain the scientific background and rationale for the investigation being reported	p.4		
Objectives	3	State specific objectives, including any prespecified hypotheses	p.4		
**Methods**					
Study Design	4	Present key elements of study design early in the paper	pp.2, 4-6		
Setting	5	Describe the setting, locations, and relevant dates, including periods of recruitment, exposure, follow-up, and data collection	pp.4-5		

For peer review only

Participants	6	*(a) Cohort study* - Give the eligibility criteria, and the sources and methods of selection of participants. Describe methods of follow-up<br>*Case-control study* - Give the eligibility criteria, and the sources and methods of case ascertainment and control selection. Give the rationale for the choice of cases and controls<br>*Cross-sectional study* - Give the eligibility criteria, and the sources and methods of selection of participants<br><br>*(b) Cohort study* - For matched studies, give matching criteria and number of exposed and unexposed<br>*Case-control study* - For matched studies, give matching criteria and the number of controls per case	pp.5-6<br>n/a<br>pp.5-6<br><br>n/a<br>n/a	RECORD 6.1: The methods of study population selection (such as codes or algorithms used to identify subjects) should be listed in detail. If this is not possible, an explanation should be provided.<br><br>RECORD 6.2: Any validation studies of the codes or algorithms used to select the population should be referenced. If validation was conducted for this study and not published elsewhere, detailed methods and results should be provided.<br><br>RECORD 6.3: If the study involved linkage of databases, consider use of a flow diagram or other graphical display to demonstrate the data linkage process, including the number of individuals with linked data at each stage.	pp.5-6<br><br>p.5<br><br>n/a
Variables	7	Clearly define all outcomes, exposures, predictors, potential confounders, and effect modifiers. Give diagnostic criteria, if applicable.	pp.5-6	RECORD 7.1: A complete list of codes and algorithms used to classify exposures, outcomes, confounders, and effect modifiers should be provided. If these cannot be reported, an explanation should be provided.	pp.5-6
Data sources/ measurement	8	For each variable of interest, give sources of data and details of methods of assessment (measurement).<br>Describe comparability of assessment methods if there is more than one group	pp.5-6		

For peer review only

Bias	9	Describe any efforts to address potential sources of bias	pp.9-10		
Study size	10	Explain how the study size was arrived at	n/a – full sample		
Quantitative variables	11	Explain how quantitative variables were handled in the analyses. If applicable, describe which groupings were chosen, and why	pp.5-6		
Statistical methods	12	(a) Describe all statistical methods, including those used to control for confounding<br>(b) Describe any methods used to examine subgroups and interactions<br>(c) Explain how missing data were addressed<br>(d) *Cohort study* - If applicable, explain how loss to follow-up was addressed<br>*Case-control study* - If applicable, explain how matching of cases and controls was addressed<br>*Cross-sectional study* - If applicable, describe analytical methods taking account of sampling strategy<br>(e) Describe any sensitivity analyses	pp.5-6<br>n/a<br>n/a<br>n/a<br>n/a<br>n/a<br>n/a		
Data access and cleaning methods		..		RECORD 12.1: Authors should describe the extent to which the investigators had access to the database population used to create the study population.	p.4

For peer review only

				RECORD 12.2: Authors should provide information on the data cleaning methods used in the study.	p.5
Linkage		..		RECORD 12.3: State whether the study included person-level, institutional-level, or other data linkage across two or more databases. The methods of linkage and methods of linkage quality evaluation should be provided.	n/a
**Results**					
Participants	13	(a) Report the numbers of individuals at each stage of the study (*e.g.*, numbers potentially eligible, examined for eligibility, confirmed eligible, included in the study, completing follow-up, and analysed)<br>(b) Give reasons for non-participation at each stage.<br>(c) Consider use of a flow diagram	pp.7-8<br>n/a<br>n/a	RECORD 13.1: Describe in detail the selection of the persons included in the study (*i.e.,* study population selection) including filtering based on data quality, data availability and linkage. The selection of included persons can be described in the text and/or by means of the study flow diagram.	pp.5-7
Descriptive data	14	(a) Give characteristics of study participants (*e.g.*, demographic, clinical, social) and information on exposures and potential confounders<br>(b) Indicate the number of participants with missing data for each variable of interest<br>(c) *Cohort study* - summarise follow-up time (*e.g.*, average and total amount)	pp.7-8<br>n/a<br>pp.7-8		
Outcome data	15	*Cohort study* - Report numbers of outcome events or summary measures over time	p.7		

For peer review only

		Case-control study - Report numbers in each exposure category, or summary measures of exposure<br>*Cross-sectional study* - Report numbers of outcome events or summary measures	n/a<br><br>p.8		
Main results	16	(a) Give unadjusted estimates and, if applicable, confounder-adjusted estimates and their precision (e.g., 95% confidence interval). Make clear which confounders were adjusted for and why they were included<br>(b) Report category boundaries when continuous variables were categorized<br>(c) If relevant, consider translating estimates of relative risk into absolute risk for a meaningful time period	pp.7-8<br><br>n/a<br><br>n/a		
Other analyses	17	Report other analyses done—e.g., analyses of subgroups and interactions, and sensitivity analyses	n/a		
**Discussion**					
Key results	18	Summarise key results with reference to study objectives	p.9		
Limitations	19	Discuss limitations of the study, taking into account sources of potential bias or imprecision. Discuss both direction and magnitude of any potential bias	pp.9-10	RECORD 19.1: Discuss the implications of using data that were not created or collected to answer the specific research question(s). Include discussion of misclassification bias, unmeasured confounding, missing data, and changing eligibility over time, as they pertain to the study being reported.	pp.9-10

For peer review only

Interpretation	20	Give a cautious overall interpretation of results considering objectives, limitations, multiplicity of analyses, results from similar studies, and other relevant evidence	p.10		
Generalisability	21	Discuss the generalisability (external validity) of the study results	p.9		
**Other Information**					
Funding	22	Give the source of funding and the role of the funders for the present study and, if applicable, for the original study on which the present article is based	p.11		
Accessibility of protocol, raw data, and programming code		..		RECORD 22.1: Authors should provide information on how to access any supplemental information such as the study protocol, raw data, or programming code.	n/a

*Reference: Benchimol EI, Smeeth L, Guttmann A, Harron K, Moher D, Petersen I, Sørensen HT, von Elm E, Langan SM, the RECORD Working Committee. The REporting of studies Conducted using Observational Routinely-collected health Data (RECORD) Statement. *PLoS Medicine* 2015; in press.

*Checklist is protected under Creative Commons Attribution (CC BY) license.

For peer review only

BMJ Open

Temporal variation in the diagnosis of resolved atrial fibrillation and the influence of performance targets on clinical coding: cohort study

Journal:	*BMJ Open*
Manuscript ID	bmjopen-2019-030454.R1
Article Type:	Original research
Date Submitted by the Author:	31-Jul-2019
Complete List of Authors:	Adderley, Nicola; University of Birmingham, Institute of Applied Health Research<br>Nirantharakumar, Krishnarajah; University of Birmingham, Institute of Applied Health Research<br>Marshall, Tom; University of Birmingham, Institute of Applied Health Research
**Primary Subject Heading**:	General practice / Family practice
Secondary Subject Heading:	Cardiovascular medicine
Keywords:	PRIMARY CARE, CARDIOLOGY, Anticoagulation < HAEMATOLOGY

SCHOLARONE™
Manuscripts

TITLE

Temporal variation in the diagnosis of resolved atrial fibrillation and the influence of performance targets on clinical coding: cohort study

AUTHORS

Nicola J Adderley[1], Krishnarajah Nirantharakumar[2], Tom Marshall[3]

[1]Research Fellow, Institute of Applied Health Research, University of Birmingham, Edgbaston, Birmingham, B15 2TT

[2]Senior Clinical Lecturer, Institute of Applied Health Research, University of Birmingham, Edgbaston, Birmingham, B15 2TT

[3]Professor of Public Health and Primary Care, Institute of Applied Health Research, University of Birmingham, Edgbaston, Birmingham, B15 2TT

Corresponding author

Tom Marshall

Address: Institute of Applied Health Research, University of Birmingham, Edgbaston, Birmingham, B15 2TT

Email: t.p.marshall@bham.ac.uk

WORD COUNT

Word count (abstract):	289
Word count (main text):	3421
Tables:	0
Figures:	3
References:	15

For peer review only

ABSTRACT

Objectives

To investigate how the introduction of performance targets for anticoagulation in atrial fibrillation (AF) affected use of the ‘resolved atrial fibrillation’ code.

Design

Retrospective cohort studies.

Setting

Data from The Health Improvement Network (THIN), a UK database of electronic patient records, from 2000 to 2016.

Participants

250,788 adult patients aged ≥18 years with a diagnosis of AF, including 14,757 with an incident diagnosis of ‘resolved AF’.

Main outcome measures

Annual and monthly incidence of ‘resolved AF’ from 2000 to 2016. Among patients with ‘resolved AF’, for each year we calculated median duration of the preceding AF diagnosis and the proportion prescribed anticoagulants prior to ‘resolved AF’.

Results

Incidence of ‘resolved AF’ increased from 5.7 to 26.3 per 1000 person-years between 2005 and the introduction of AF performance targets in 2006. Compared to the years prior to the introduction of the performance targets, incidence has remained higher in every year since their implementation. Since 2007, monthly incidence has been highest between January and March. Between 2005 and 2006, median duration between AF and ‘resolved AF’ diagnoses increased from 276 days (9 months) to 1343 days (3 years 8 months). Among ‘resolved AF’ patients with CHA_2DS_2-VASc score ≥1, 81.9% (95%CI 81.1 to 82.6) had no current anticoagulant prescription, and 62.3% (95%CI 61.4 to 63.2) had no record of any anticoagulant prescription.

Conclusion

The introduction of AF performance targets was followed by a large increase in use of the ‘resolved AF’ code, particularly in the months immediately before practices make their anticoagulant performance target submissions. Although most AF patients are prescribed anticoagulants, few patients diagnosed with ‘resolved AF’ are prescribed anticoagulants and most have never been prescribed them. Untreated patients are much more likely to be coded as having ‘resolved AF’.

For peer review only - http://bmjopen.bmj.com/site/about/guidelines.xhtml

Strengths and limitations of this study

- Analysis was performed in a large primary care dataset which is generalisable to the UK population and included more than a quarter of a million patients with atrial fibrillation (AF).
- Data was derived from routinely clinical data which is used by general practitioners for clinical decision-making.
- The study assessed the impact of the introduction of AF into the Quality and Outcomes Framework on the use of the 'resolved AF' clinical code.
- Use and interpretation of the 'resolved AF' code is likely to vary between general practitioners and practices.
- The primary care dataset contains no direct information on general practitioners' reasons for assigning a 'resolved AF' code; possible influencing factors must therefore be inferred from explorations of temporal variation, patient diagnostic information and anticoagulant prescribing.

For peer review only

For peer review only - http://bmjopen.bmj.com/site/about/guidelines.xhtml

INTRODUCTION

Atrial fibrillation (AF) is a common cardiac arrhythmia associated with increased risk of stroke and transient ischaemic attack (TIA); this increased risk is attenuated by treatment with anticoagulants.[1,2,3] AF may be categorised as resolved if normal heart rhythm is restored. However, AF may recur after apparent resolution. [4,5] Evidence shows that patients diagnosed as having 'resolved AF' continue to be at increased risk of stroke/TIA; from 2013 to 2016, risk in patients with 'resolved AF' was found to be the same as that in patients with ongoing AF.[6]

Factors influencing clinicians to make a diagnosis of 'resolved AF' are unclear. Research has demonstrated that the prevalence of the AF diagnosed clinical code in UK general practice increased significantly after 2006 and has remained comparatively high since.[6] The Quality and Outcomes Framework (QOF) is a scheme to improve the clinical quality of care for chronic diseases. General practices keep a register of patients with particular chronic diseases and are paid an incentive for achieving performance targets for the management of patients on the register. AF was introduced into QOF in 2006 with an incentive payment for ensuring that more than a specified percentage of patients received drugs for stroke prevention.[7] The increase in prevalence of 'resolved AF' after 2006 suggests QOF may have contributed to the increase in 'resolved AF' diagnoses. There was no corresponding jump in the recorded prevalence of AF at this time.[8] From April 2006, general practices were required to maintain a register of patients with AF and to record whether eligible patients were prescribed anticoagulants or antiplatelets. In 2012, the AF QOF indicators were updated to include an assessment of stroke risk and to require patients with a high stroke risk to be treated with anticoagulants (not antiplatelets).[9]

We hypothesised that the introduction of AF into QOF had an impact on the use of the 'resolved AF' code. The aim of this analysis, therefore, was to use information available in routinely collected primary care data to explore this hypothesis by investigating variation in the use of the 'resolved AF' clinical code over time and across different practices, and to investigate other factors which may influence general practitioners to assign a diagnosis of 'resolved AF'. The specific questions addressed were:

1. What is the annual incidence of 'resolved AF' diagnoses and did incidence increase with the introduction of AF into QOF?
2. Since the introduction of AF into QOF, is a diagnosis of 'resolved AF' more likely to be recorded in the months of January to March, immediately prior to the practice QOF submission?
3. Is there a difference in the duration of AF diagnosis in patients diagnosed as having 'resolved AF' before and after the introduction of AF into QOF?
4. Are patients prescribed anticoagulants before their 'resolved AF' diagnosis?
5. How much variation exists between general practices in use of the 'resolved AF' code?

Evidence indicating that use of the 'resolved AF' code is substantially driven by QOF reporting would support the recommendation that patients with 'resolved AF' be included in QOF AF registers and receive ongoing AF management,[6] or that the 'resolved AF' clinical code be withdrawn.

For peer review only - http://bmjopen.bmj.com/site/about/guidelines.xhtml

METHODS

Data source

Datasets were extracted from The Health Improvement Network (THIN), a database of electronic primary care records from UK general practices using Vision software. The version of the database from which study datasets were derived included data for approximately 14 million patients at over 640 practices. THIN comprises coded data on patient demographics, diagnoses, prescriptions issued in primary care, consultations and investigations. Data on all prescriptions issued in primary care are recorded in THIN; diagnoses that are part of the QOF are well recorded.

Population

General practices were eligible for participation from the later of the practice acceptable mortality recording (AMR) date,[10] Vision installation date plus one year, and the study start date (1 year prior to the first index/census date).

All adult patients aged 18 years and over with a recorded diagnosis of atrial fibrillation and registered for at least 365 days before the index/census date were eligible for inclusion. AF was defined by a record of a relevant clinical (Read) code.

Study design

A retrospective cohort study from 1st January 2000 to 31st December 2016 was carried out. Index date was the latest of the following two dates: one year after the patient registered with the practice or the date of diagnosis of AF.

To determine incidence of 'resolved AF' among patients with AF, eligible patients were followed up from the index date until the earliest of the following: patient left the practice/transferred out, death, study end date, most recent data upload from practice, or a diagnosis of 'resolved AF'. Patients with a record of 'resolved AF' at study entry were excluded. 'Resolved AF' was defined as a record of the relevant clinical (Read) code (212R.00 'Atrial fibrillation resolved').[6]

To explore temporal variation in AF duration and anticoagulant prescribing preceding a diagnosis of 'resolved AF', a cohort restricted to patients with a diagnosis of 'resolved AF' during the study period was used. Eligible patients were followed up until the earliest of the following: patient left practice/transferred out, death, study end date, most recent data upload from practice, or an outcome event.

To explore practice-level variation in use of the 'resolved AF' clinical code, a cross-sectional study was carried out on 1st December 2016.

For peer review only - http://bmjopen.bmj.com/site/about/guidelines.xhtml

Analysis

Annual incidence of 'resolved AF'

Annual incidence rates of a 'resolved AF' diagnosis among AF patients were calculated for each year from 2000 to 2016 by dividing the number of patients with a new (first) record of 'resolved AF' (numerator) by the total number of person-years at risk (denominator) for the given year.

Monthly variation in use of the 'resolved AF' code pre- and post-QOF

To investigate the impact of QOF on the distribution of 'resolved AF' coding throughout the year, monthly incidence of 'resolved AF' diagnoses (in each month from January to December) was calculated in the pre-QOF period (2000 to 2005), in 2006 and 2007, and in the post-QOF period (2008 to 2016). Monthly incidence was calculated separately for 2006 and 2007 as annual incidence of 'resolved AF' in this period, the years of and immediately following the introduction of AF into QOF, was found to be substantially higher than in subsequent years.

In the post-QOF period (2007 onwards), Poisson regression was used to calculate crude and adjusted incidence rate ratios of stroke/transient ischaemic attack (TIA) in patients with a 'resolved AF' diagnosis recorded in January to March compared to April to December, in order to explore any possible differences in disease severity between patients coded as resolved at different times of the year. The adjusted model included the following covariates: age, sex, CHA_2DS_2-VASc score (categorised as 0, 1 ≥2) and prescription of anticoagulant medication at the time of the 'resolved AF' diagnosis.

'Resolved AF' cohort

The following analyses were restricted to patients with a record of 'resolved AF'.

Duration of AF diagnosis

To explore variation over time in duration of AF diagnosis in patients with 'resolved AF', median (interquartile range, IQR) duration of time between diagnosis of AF (earliest recorded Read code) and first record of a 'resolved AF' code was calculated for each year in patients with a 'resolved AF' code.

Anticoagulant prescribing

To explore prescribing of anticoagulants to patients with a diagnosis of 'resolved AF', the proportion of patients on anticoagulant treatment at the time of diagnosis (current treatment, prescribed up to 90 days prior to 'resolved AF' record), 0 to 90 days, and 91 to 180 days after the 'resolved AF' diagnosis were calculated with 95% CIs for proportions in 1) all 'resolved AF' patients and 2) patients with a CHA_2DS_2-VASc score ≥1 (eligible for anticoagulant treatment). The proportion of 'resolved AF' patients with a CHA_2DS_2-VASc score ≥1 who had never been prescribed anticoagulants was also calculated. Trends over time were explored by calculating the proportions for each year between 2000 and 2016.

For peer review only - http://bmjopen.bmj.com/site/about/guidelines.xhtml

Cross-sectional analysis

Practice-level variation in use of 'resolved AF' code

Variation in use of the 'resolved AF' code by general practice in 2016 was assessed by plotting the percentage of AF patients with any record of a 'resolved AF' code (ever) at a given practice against the number of AF patients at the practice. Upper and lower control limits (within 3 standard deviations of the mean) were calculated.

Definitions of variables

AF, 'resolved AF', and stroke/TIA were defined by the presence of a clinical code; the absence of a clinical code was taken to indicate no diagnosis. The clinical code lists used have been utilised in a number of previous AF studies[6,8,11,12,13] and include all codes used in QOF.[14]

CHA_2DS_2-VASc scores were calculated by adding 1 point each for a history of congestive heart failure (HF), hypertension, diabetes (DM), vascular disease, age 65-74 years and female sex (if another risk factor was present, otherwise 0), and 2 points for age ≥75 and a history of stroke/TIA. HF, hypertension, DM and vascular disease were defined by a relevant clinical code.

Anticoagulants included warfarin, parenteral anticoagulants, other vitamin K antagonists, and novel/non-vitamin K oral anticoagulants.

All statistical analyses were performed in Stata IC version 14.2.

Patient involvement

Patients were not involved in the research.

RESULTS

Annual incidence of 'resolved AF'

A total of 250,788 patients with AF contributing 1,037,858 person-years were included in the analysis; 14,757 patients had an incident diagnosis of 'resolved AF'. Mean (SD) age was 74.6 (12.1) years; 52.6% pf patients were male; median (IQR) follow-up was 3.1 (1.2-6.1) years.

Incidence of the atrial fibrillation (AF) resolved code in patients with AF showed a sharp rise in 2006 (Figure 1), at which time AF was introduced into QOF, rising from 5.7 per 1000 person-years in 2005 to 26.3 per 1000 person-years in 2006. Incidence peaked at 28.6 per 1000 person-years in 2007; it declined thereafter, before rising again to 19.5 per 1000 person-years in 2012-13, when further changes were made to the QOF AF requirements. Since 2013 the incidence has declined.

Monthly variation in use of the 'resolved AF' code

Prior to the introduction of AF into QOF (January 2000 to March 2006), incidence of the 'resolved AF' code remained relatively constant across the 12 months of the year, including the 3 months

For peer review only - http://bmjopen.bmj.com/site/about/guidelines.xhtml

immediately prior to the introduction of AF into QOF (January to March 2006), with monthly incidence varying between 3.2 and 7.2 per 1000 person-years (Figure 2). From April 2006 and for the subsequent 12 months, incidence of the code steadily increased, reaching a peak of 70.2 per 1000 person-years in January 2007. From 2007 onwards (post-QOF), incidence of the 'resolved AF' code has been highest between the months of January and March, the 3 months immediately preceding QOF report submission. In the post-QOF period (2008 to 2016) incidence is higher in every month of the year relative to the same month in the pre-QOF period.

From 2007 onwards, 245 patients diagnosed with 'resolved AF' in January to March and 358 patients diagnosed in April to December had a stroke. Crude incidence rates were 12.4 and 13.8 per 1000 person-years, respectively. Among patients who received a diagnosis of 'resolved AF' after the introduction of AF into QOF (2007 onwards), there was no difference in incidence of stroke/TIA in patients who were assigned the code between January and March compared to those given the code later in the year: crude IRR 0.90 (95% CI 0.76 to 1.06), adjusted IRR 0.98 (95% CI 0.83 to 1.15).

'Resolved AF' cohort

14,863 patients with a record of 'resolved AF' were included in the cohort from 2000 to 2016. Median (IQR) age was 70.7 (59.6-79.6); 58.1% of patients were male. 11,479 (77.2%) patients had a CHA_2DS_2-VASc score ≥1. Median (IQR) follow-up was 3.8 (1.9-6.8) years. 3,384 (22.8%), 1,737 (11.7%) and 9,742 (65.5%) patients had a CHA_2DS_2-VASc score of 0, 1 or ≥2, respectively.

Duration of time between diagnosis of AF and use of the 'resolved AF' code

Median duration of time between diagnosis of AF and first recording of a 'resolved AF' code remained between several months and approximately a year (varying from 69 to 335 days) between 2000 and 2005. In 2006 there was a sharp rise in median duration from 276 days (9 months) in 2005 to 1343 days (3 years 8 months) in 2006. This indicates that in 2006 more than half of patients who were assigned a 'resolved AF' code had been diagnosed over 3 ½ years earlier. Median duration then declined for several years, before rising again to more than 1000 days in 2012-13.

Sequence of events in relation to anticoagulant prescribing in 'resolved AF' patients

Few patients were still on anticoagulants when the 'resolved AF' code was recorded. In the cohort of 'resolved AF' patients (2000 to 2016), 17.3% (95% CI 16.7 to 17.9) had a current prescription at the time of 'resolved AF' recording (up to 90 days prior), with 82.7% (95% CI 82.1 to 83.3) not being prescribed anticoagulant treatment. There was no correlation between anticoagulant prescribing and CHA_2DS_2-VASc category: 14.6%, 25.6% and 16.8% of patients with scores of 0, 1 and ≥2, respectively, were prescribed anticoagulants. Up to 90 days following the 'resolved AF' diagnosis, 9.8% (95% CI 9.3 to 10.3) of patients were still being prescribed anticoagulants. By 91 to 180 days after 'resolved AF', 8.7% (95% CI 8.3 to 9.2) had a prescription for anticoagulants.

Among 'resolved AF' patients with a CHA_2DS_2-VASc score ≥1, 18.1% (95% CI 17.4 to 18.9) had a current prescription for anticoagulants, while 81.9% (95% CI 81.1 to 82.6) had no current prescription. 10.5% (95% CI 10.0 to 11.1) and 9.7% (95% CI 9.2 to 10.3) had prescriptions up to 90 days and 91 to 180 days following the 'resolved AF' diagnosis respectively. The proportion of

For peer review only - http://bmjopen.bmj.com/site/about/guidelines.xhtml

'resolved AF' patients prescribed anticoagulants shortly before and after recording of the 'resolved AF' code varied slightly over time, with a notable drop in 2006 to 9.8% (95% CI 8.5 to 11.4), decreasing from 25.2% (95% CI 20.6 to 30.3) in 2005.

62.3% (95% CI 61.4 to 63.2) of 'resolved AF' patients with a CHA_2DS_2-VASc score ≥1 had no record of an anticoagulant prescription. Among the cohort of patients whose first record of AF was after registration with the practice (n=13,307), 60.6% (95% CI 59.6 to 61.5) had never been prescribed anticoagulants; this proportion varied slightly over time, reaching a peak of 70.2% in 2006 and a low of 51.3% in 2016.

Practice-level variation

787 practices with a total of 1,167,771 patients with AF were included in the analysis from 2000 to 2016. 443 practices with a total of 69,262 patients with AF, of whom 7,261 had a record of 'resolved AF', were included in the analysis in 2016.

Variation in use of the 'resolved AF' code between general practices

The proportion of AF patients with a record of 'resolved AF' varied between practices, ranging from 0% to 43% in 2016. The majority of practices fell within the acceptable range (between the upper (UCL) and lower (LCL) control limits) based on the size of the practice AF population, although a number of practices fell outside this range: 54 (12.2%) practices above the UCL and 30 (6.8%) below the LCL (Figure 3). In 2016, 3 practices with more than 100 patients with AF assigned a 'resolved AF' code to none of these patients, while 10 practices assigned a 'resolved AF' code to more than 25% of patients with AF.

Similar patterns in variation were observed in the year immediately after the introduction of AF into QOF (2007): the proportion of patients with 'resolved AF' ranged from 0% to 40%, with 61 (13.8%) practices above the UCL and 30 (6.8%) below the LCL. In 2005, immediately before the introduction of AF into QOF, there was slightly less variation: the proportion of patients with 'resolved AF' ranged from 0% to 30%, with 39 (8.8%) practices above the UCL. None were below the LCL, which was low due to the smaller average number of patients with 'resolved AF'.

For peer review only

DISCUSSION

Incidence of 'resolved AF' rose dramatically in 2006 immediately following the introduction of AF into the Quality and Outcomes Framework (QOF).[7] Incidence peaked the following year at 28.6 per 1000 person-years, showing a five-fold increase compared to the incidence prior to QOF. There was a further, smaller, peak in 'resolved AF' incidence in 2012-13, following a change in the QOF AF indicators to introduce a stroke risk assessment indicator and to change the requirements for the anticoagulation indicator.[9] A corresponding rise in the prevalence of 'resolved AF' among patients with AF, from 2.3% in 2005 to 6.4% in 2007 and a high of 9.2% in 2013, has been reported previously.[6]

Since the introduction of AF into QOF, the majority of 'resolved AF' codes have been recorded between the months of January and March, immediately prior to QOF report submission by general

For peer review only - http://bmjopen.bmj.com/site/about/guidelines.xhtml

practices. Prior to this, 'resolved AF' codes were recorded throughout the year with little monthly variation in incidence. There is no difference in stroke/TIA rates in patients diagnosed as having 'resolved AF' between January and March compared to those diagnosed later in the year; patients with AF who are diagnosed as resolved immediately prior to QOF do not have a different/lower risk of stroke/TIA.

Immediately following the introduction of AF into QOF, there was a dramatic rise in median duration between AF and 'resolved AF' diagnoses, with a further peak at the time of changes to QOF in 2012-13. At these time points, patients designated as having 'resolved AF' had been diagnosed with AF several years previously (median 3 years and 8 months in 2006) compared around one year prior to QOF (9 months in 2005).

Almost two thirds of patients with 'resolved AF' and a CHA_2DS_2-VASc score ≥1 had never been prescribed anticoagulants. In 2016, 79.5% of patients with 'resolved AF' and a CHA_2DS_2-VASc score ≥1 were not prescribed anticoagulants at the time of their 'resolved AF' diagnosis, made up of 53.5% who had never been prescribed anticoagulants and 26.0% who had previously been prescribed anticoagulants but had subsequently discontinued. By contrast, only 25-30% of patients with ongoing AF and a CHA_2DS_2-VASc score ≥1 were not prescribed anticoagulants in 2016.[8,15] This suggests that patients with AF who are not prescribed anticoagulants may be more likely to be assigned a 'resolved AF' code. Furthermore, recent evidence indicates that patients with a diagnosis of 'resolved AF' remain at increased risk of stroke/TIA and may therefore benefit from continued anticoagulant prophylaxis.[6]

Use of the 'resolved AF' code varies between practices. Some practices with large numbers of AF patients use the code for very few patients, while others assign the code to more than a quarter of AF patients.

Strengths and limitations

This analysis was performed in a large general practice dataset which is generalisable to the UK population. Data was derived from routinely clinical data which is used by general practitioners for clinical decision-making. The use and interpretation of the 'resolved AF' clinical code is likely to vary between general practitioners and practices. The primary care dataset contains no direct information on general practitioners' reasons for assigning a 'resolved AF' code; possible influencing factors have therefore been inferred from explorations of temporal variation, patient diagnostic information and anticoagulant prescribing. In order to better understand the factors motivating a diagnosis of 'resolved AF', a qualitative study and consultation with practicing clinicians would be required. Anticoagulation rates may be underestimated if treatment is managed entirely in secondary care; however, the majority of anticoagulants are prescribed in primary care.

Conclusions

Use of the 'resolved AF' code remains common. Most patients eligible for anticoagulant treatment who were assigned a 'resolved AF' code were never prescribed anticoagulants, and very few patients were still taking anticoagulants when the 'resolved AF' code was recorded. Those diagnosed as having 'resolved AF' are no longer included in the AF register for QOF; this has the effect of improving the practice's apparent performance in the QOF. Incidence of the 'resolved AF' clinical code increased markedly when AF was introduced into QOF in 2006 and increased again when

For peer review only - http://bmjopen.bmj.com/site/about/guidelines.xhtml

further changes were made to the QOF incentive scheme in 2012. Since 2006, incidence of the 'resolved AF' code has been highest in the months shortly before practices make their QOF submissions. Previous evidence demonstrated patients with a diagnosis of 'resolved AF' remain at increased risk of stroke/TIA and are therefore likely to benefit from anticoagulant prophylaxis. We therefore recommend that patients with 'resolved AF' should be included when determining whether practices meet QOF clinical performance targets.

For peer review only

For peer review only - http://bmjopen.bmj.com/site/about/guidelines.xhtml

FIGURE LEGENDS

Figure 1. Annual incidence of resolved atrial fibrillation in patients with AF 2000-2016.

Figure 2. Incidence of the 'resolved AF' code by month of recording, before, during and after the introduction of AF into the Quality and Outcomes Framework (QOF).

Figure 3. Funnel plot showing variation in use of the 'resolved AF' code by practice in 2016.

ETHICAL APPROVAL

The THIN data collection scheme and research carried out using THIN data were approved by the NHS South-East Multicentre Research Ethics Committee (MREC) in 2003; under the terms of this approval, studies must undergo independent scientific review. Approval for these analyses was obtained from the Scientific Review Committee (for the use of THIN data) in April 2015 (15THIN021) and September 2017 (SRC reference number 17THIN082).

COMPETING INTERESTS

All authors have completed the ICMJE uniform disclosure form at http://www.icmje.org/coi_disclosure.pdf and declare: NA and TM report a grant from the National Institute for Health Research (NIHR) Collaboration for Leadership in Applied Health Research and Care West Midlands during the conduct of the study; KN reports funding from AstraZeneca and fees from Sanofi and Boehringer Ingelheim outside the submitted work. Authors declare no other financial relationships with any organisations that might have an interest in the submitted work in the previous three years; and no other relationships or activities that could appear to have influenced the submitted work.

CONTRIBUTORS

NA, KN and TM designed the study. KN undertook data extraction. NA designed and performed the analyses. NA wrote the first draft of the paper, which was revised in collaboration with TM and KN. NA acts as guarantor.

FUNDING

NJA and TM were funded by the NIHR Collaboration for Leadership in Applied Health Research and Care West Midlands initiative (NIHR CLAHRC-WM). This paper presents independent research and the views expressed are those of the author(s) and not necessarily those of the NHS, the NIHR or the Department of Health.

The funders had no role in study design, data collection and analysis, decision to publish, or preparation of the manuscript.

For peer review only

TRANSPARENCY

The lead author (the manuscript's guarantor) affirms that the manuscript is an honest, accurate, and transparent account of the study being reported; that no important aspects of the study have been omitted; and that any discrepancies from the study as planned (and, if relevant, registered) have been explained.

DATA SHARING STATEMENT

Dataset is not available.

EXCLUSIVE LICENCE

I, the Submitting Author has the right to grant and does grant on behalf of all authors of the Work (as defined in the below author licence), an exclusive licence and/or a non-exclusive licence for contributions from authors who are: i) UK Crown employees; ii) where BMJ has agreed a CC-BY licence shall apply, and/or iii) in accordance with the terms applicable for US Federal Government officers or employees acting as part of their official duties; on a worldwide, perpetual, irrevocable, royalty-free basis to BMJ Publishing Group Ltd ("BMJ") its licensees and where the relevant Journal is co-owned by BMJ to the co-owners of the Journal, to publish the Work in BMJ Open and any other BMJ products and to exploit all rights, as set out in our licence.

For peer review only

For peer review only - http://bmjopen.bmj.com/site/about/guidelines.xhtml

REFERENCES

[1] Antithrombotic Trialists' Collaboration. Collaborative meta-analysis of randomised trials of antiplatelet therapy for prevention of death, myocardial infarction, and stroke in high risk patients. *BMJ* 2002; 324:71–86.

[2] Aguilar MI and Hart R. Oral anticoagulants for preventing stroke in patients with non-valvular atrial fibrillation and no previous history of stroke or transient ischemic attacks. Cochrane Database of Systematic Reviews 2005, Issue 3. Art. No.: CD001927. doi: 10.1002/14651858.CD001927.pub2.

[3] Aguilar MI, Hart R and Pearce LA. Oral anticoagulants versus antiplatelet therapy for preventing stroke in patients with non-valvular atrial fibrillation and no history of stroke or transient ischemic attacks. *Cochrane Database of Systematic Reviews* 2007, Issue 3. Art. No.: CD006186. doi: 10.1002/14651858.CD006186.pub2.

[4] Wynn GJ, El-Kadri M, Haq I, Das M, Modi S, Snowdon R, Hall M, Waktare JE, Todd DM, Gupta D. Long-term outcomes after ablation of persistent atrial fibrillation: an observational study over 6 years. Open Heart. 2016;3:e000394. doi: 10.1136/openhrt-2015-000394

[5] Militaru C, Donoiu I. Atrial Fibrillation Recurrence Predictors after Conversion to Sinus Rhythm Curr Health Sci J. 2014 Apr-Jun; 40(2): 129–133. doi:10.12865/CHSJ.40.02.09

[6] Adderley NJ, NIrantharakumar K, Marshall T. Risk of stroke and transient ischaemic attack in patients with a diagnosis of resolved atrial fibrillation: retrospective cohort studies. BMJ 2018. doi:10.1136/bmj.k1717

[7] NHS Employers and General Practitioners Committee. Revisions to the GMS contract 2006/07. Delivering investment in general practice. NHS Employers: London 2006.

[8] Adderley NJ, Ryan R, Nirantharakumar K, Marshall T. Prevalence and treatment of atrial fibrillation in UK general practice from 2000 to 2016. Heart 2018. doi:10.1136/heartjnl-2018-312977

[9] NHS Employers and General Practitioners Committee. Quality and Outcomes Framework for 2012/13. Guidance for PCOs and practices. NHS Employers: London 2012.

[10] Maguire A, Blak BT, Thompson M. The importance of defining periods of complete mortality reporting for research using automated data from primary care. *Pharmacoepidemiol Drug Saf* 2009;18:76e83.

[11] Cowan C, Healicon R, Robson I, et al. The use of anticoagulants in the management of atrial fibrillation among general practices in England. *Heart* 2013;99:1166–72. doi: 10.1136/heartjnl-2012-303472

[12] Adderley N, Ryan R, Marshall T. The role of contraindications in prescribing anticoagulants to patients with atrial fibrillation: a cross-sectional analysis of primary care data in the UK. *Br J Gen Prac* 2017. doi: 10.3399/bjgp17X691685

[13] Isaew A, Adderley NJ, Ryan R, Fitzmaurice D, Marshall T. The treatment of paroxysmal atrial fibrillation in UK primary care. *Heart* 2017;103:1502-7. doi: 10.1136/heartjnl-2016-310927

[14] NHS England. New GMS Contract QOF Implementation. Dataset and Business Rules. Atrial Fibrillation Indicator Set. Leeds: Health and Social Care Information Centre 2016.

[15] Lacoin L, Lumley M, Ridha E, Pereira M, McDonald L, Ramagopalan S, et al. Evolving landscape of stroke prevention in atrial fibrillation within the UK between 2012 and 2016: a cross-sectional analysis study using CPRD. BMJ Open 2017;7:e015363. doi:10.1136/bmjopen-2016-015363

For peer review only

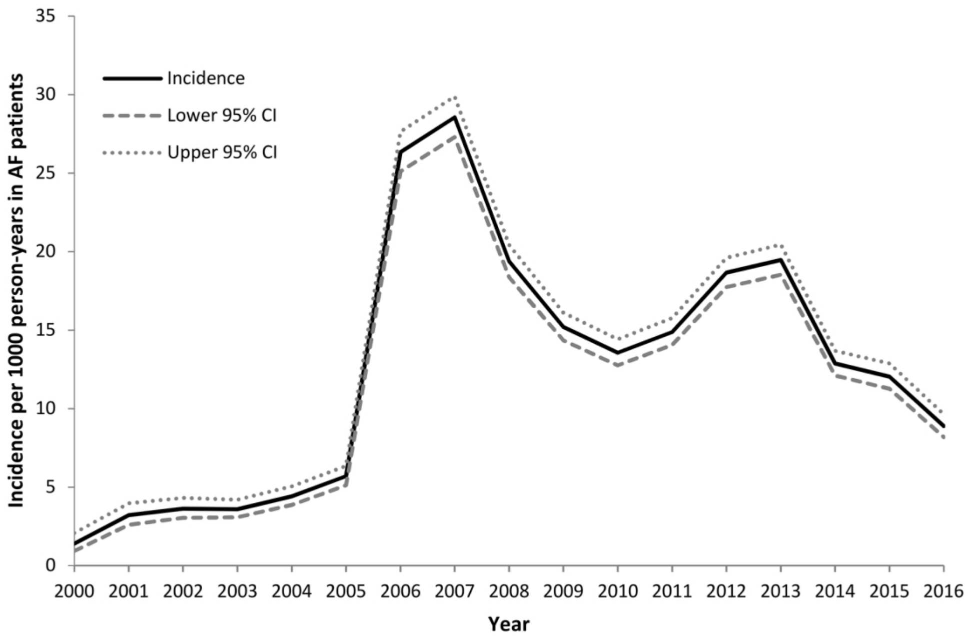

Figure 1. Annual incidence of resolved atrial fibrillation in patients with AF 2000-2016.

157x105mm (300 x 300 DPI)

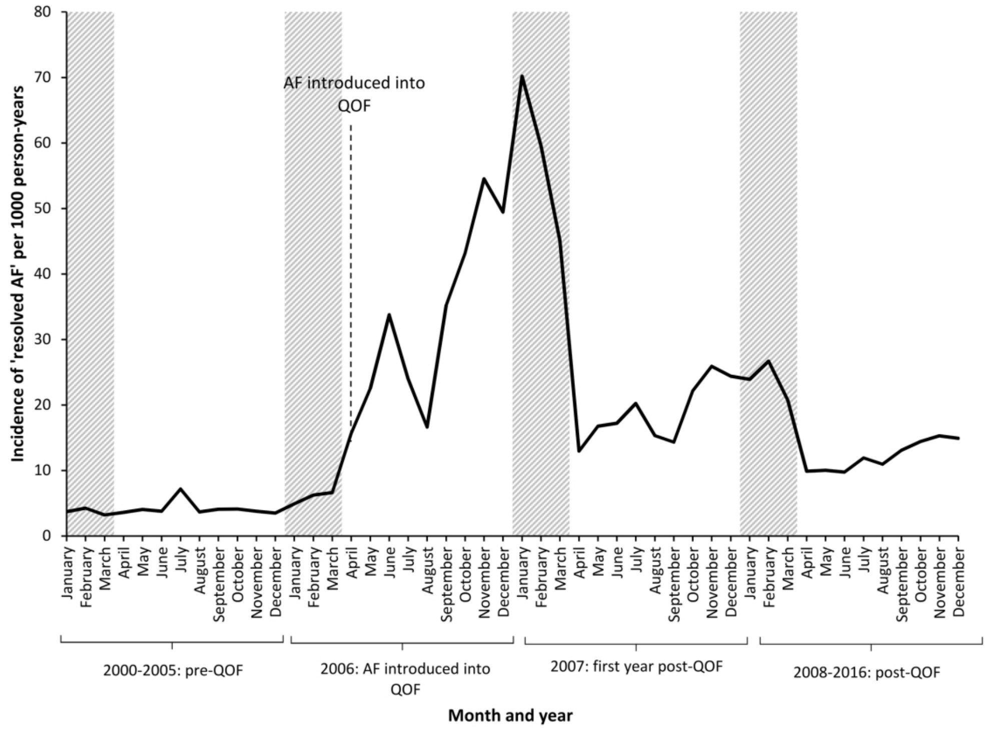

Figure 2. Incidence of the 'resolved AF' code by month of recording, before, during and after the introduction of AF into the Quality and Outcomes Framework (QOF).

188x138mm (300 x 300 DPI)

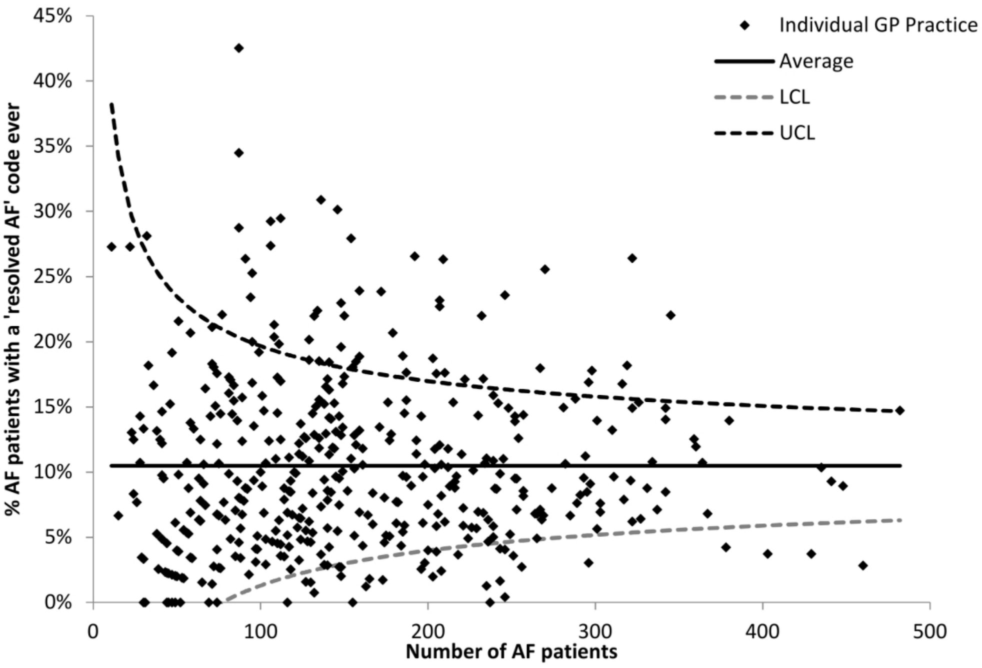

Figure 3. Funnel plot showing variation in use of the 'resolved AF' code by practice in 2016.

155x104mm (300 x 300 DPI)

The RECORD statement – checklist of items, extended from the STROBE statement, that should be reported in observational studies using routinely collected health data.

	Item No.	STROBE items	Location in manuscript where items are reported	RECORD items	Location in manuscript where items are reported
**Title and abstract**					
	1	(a) Indicate the study's design with a commonly used term in the title or the abstract (b) Provide in the abstract an informative and balanced summary of what was done and what was found	p.3	RECORD 1.1: The type of data used should be specified in the title or abstract. When possible, the name of the databases used should be included.<br><br>RECORD 1.2: If applicable, the geographic region and timeframe within which the study took place should be reported in the title or abstract.<br><br>RECORD 1.3: If linkage between databases was conducted for the study, this should be clearly stated in the title or abstract.	p.3<br><br>p.3<br><br>n/a
**Introduction**					
Background rationale	2	Explain the scientific background and rationale for the investigation being reported	p.4		
Objectives	3	State specific objectives, including any prespecified hypotheses	p.4		
**Methods**					
Study Design	4	Present key elements of study design early in the paper	pp.2, 4-6		
Setting	5	Describe the setting, locations, and relevant dates, including periods of recruitment, exposure, follow-up, and data collection	pp.4-5		

For peer review only

Participants	6	*(a) Cohort study* - Give the eligibility criteria, and the sources and methods of selection of participants. Describe methods of follow-up<br>*Case-control study* - Give the eligibility criteria, and the sources and methods of case ascertainment and control selection. Give the rationale for the choice of cases and controls<br>*Cross-sectional study* - Give the eligibility criteria, and the sources and methods of selection of participants<br><br>*(b) Cohort study* - For matched studies, give matching criteria and number of exposed and unexposed<br>*Case-control study* - For matched studies, give matching criteria and the number of controls per case	pp.5-6<br>n/a<br>pp.5-6<br><br>n/a<br>n/a	RECORD 6.1: The methods of study population selection (such as codes or algorithms used to identify subjects) should be listed in detail. If this is not possible, an explanation should be provided.<br><br>RECORD 6.2: Any validation studies of the codes or algorithms used to select the population should be referenced. If validation was conducted for this study and not published elsewhere, detailed methods and results should be provided.<br><br>RECORD 6.3: If the study involved linkage of databases, consider use of a flow diagram or other graphical display to demonstrate the data linkage process, including the number of individuals with linked data at each stage.	pp.5-6<br><br>p.5<br><br>n/a
Variables	7	Clearly define all outcomes, exposures, predictors, potential confounders, and effect modifiers. Give diagnostic criteria, if applicable.	pp.5-6	RECORD 7.1: A complete list of codes and algorithms used to classify exposures, outcomes, confounders, and effect modifiers should be provided. If these cannot be reported, an explanation should be provided.	pp.5-6
Data sources/ measurement	8	For each variable of interest, give sources of data and details of methods of assessment (measurement).<br>Describe comparability of assessment methods if there is more than one group	pp.5-6		

For peer review only

Bias	9	Describe any efforts to address potential sources of bias	pp.9-10		
Study size	10	Explain how the study size was arrived at	n/a – full sample		
Quantitative variables	11	Explain how quantitative variables were handled in the analyses. If applicable, describe which groupings were chosen, and why	pp.5-6		
Statistical methods	12	(a) Describe all statistical methods, including those used to control for confounding<br>(b) Describe any methods used to examine subgroups and interactions<br>(c) Explain how missing data were addressed<br>(d) *Cohort study* - If applicable, explain how loss to follow-up was addressed<br>*Case-control study* - If applicable, explain how matching of cases and controls was addressed<br>*Cross-sectional study* - If applicable, describe analytical methods taking account of sampling strategy<br>(e) Describe any sensitivity analyses	pp.5-6<br>n/a<br>n/a<br>n/a<br>n/a<br>n/a<br>n/a		
Data access and cleaning methods		..		RECORD 12.1: Authors should describe the extent to which the investigators had access to the database population used to create the study population.	p.4

For peer review only

				RECORD 12.2: Authors should provide information on the data cleaning methods used in the study.	p.5
Linkage		..		RECORD 12.3: State whether the study included person-level, institutional-level, or other data linkage across two or more databases. The methods of linkage and methods of linkage quality evaluation should be provided.	n/a
**Results**					
Participants	13	(a) Report the numbers of individuals at each stage of the study (*e.g.*, numbers potentially eligible, examined for eligibility, confirmed eligible, included in the study, completing follow-up, and analysed)<br>(b) Give reasons for non-participation at each stage.<br>(c) Consider use of a flow diagram	pp.7-8<br>n/a<br>n/a	RECORD 13.1: Describe in detail the selection of the persons included in the study (*i.e.,* study population selection) including filtering based on data quality, data availability and linkage. The selection of included persons can be described in the text and/or by means of the study flow diagram.	pp.5-7
Descriptive data	14	(a) Give characteristics of study participants (*e.g.*, demographic, clinical, social) and information on exposures and potential confounders<br>(b) Indicate the number of participants with missing data for each variable of interest<br>(c) *Cohort study* - summarise follow-up time (*e.g.*, average and total amount)	pp.7-8<br>n/a<br>pp.7-8		
Outcome data	15	*Cohort study* - Report numbers of outcome events or summary measures over time	p.7		

For peer review only

		Case-control study - Report numbers in each exposure category, or summary measures of exposure<br>*Cross-sectional study* - Report numbers of outcome events or summary measures	n/a<br><br><br><br>p.8		
Main results	16	(a) Give unadjusted estimates and, if applicable, confounder-adjusted estimates and their precision (e.g., 95% confidence interval). Make clear which confounders were adjusted for and why they were included<br>(b) Report category boundaries when continuous variables were categorized<br>(c) If relevant, consider translating estimates of relative risk into absolute risk for a meaningful time period	pp.7-8<br><br><br><br><br><br><br>n/a<br><br><br>n/a		
Other analyses	17	Report other analyses done—e.g., analyses of subgroups and interactions, and sensitivity analyses	n/a		
**Discussion**					
Key results	18	Summarise key results with reference to study objectives	p.9		
Limitations	19	Discuss limitations of the study, taking into account sources of potential bias or imprecision. Discuss both direction and magnitude of any potential bias	pp.9-10	RECORD 19.1: Discuss the implications of using data that were not created or collected to answer the specific research question(s). Include discussion of misclassification bias, unmeasured confounding, missing data, and changing eligibility over time, as they pertain to the study being reported.	pp.9-10

For peer review only

Interpretation	20	Give a cautious overall interpretation of results considering objectives, limitations, multiplicity of analyses, results from similar studies, and other relevant evidence	p.10		
Generalisability	21	Discuss the generalisability (external validity) of the study results	p.9		
**Other Information**					
Funding	22	Give the source of funding and the role of the funders for the present study and, if applicable, for the original study on which the present article is based	p.11		
Accessibility of protocol, raw data, and programming code		..		RECORD 22.1: Authors should provide information on how to access any supplemental information such as the study protocol, raw data, or programming code.	n/a

*Reference: Benchimol EI, Smeeth L, Guttmann A, Harron K, Moher D, Petersen I, Sørensen HT, von Elm E, Langan SM, the RECORD Working Committee. The REporting of studies Conducted using Observational Routinely-collected health Data (RECORD) Statement. *PLoS Medicine* 2015; in press.

*Checklist is protected under Creative Commons Attribution (CC BY) license.

For peer review only

BMJ Open

Temporal variation in the diagnosis of resolved atrial fibrillation and the influence of performance targets on clinical coding: cohort study

Journal:	*BMJ Open*
Manuscript ID	bmjopen-2019-030454.R2
Article Type:	Original research
Date Submitted by the Author:	26-Sep-2019
Complete List of Authors:	Adderley, Nicola; University of Birmingham, Institute of Applied Health Research<br>Nirantharakumar, Krishnarajah; University of Birmingham, Institute of Applied Health Research<br>Marshall, Tom; University of Birmingham, Institute of Applied Health Research
**Primary Subject Heading**:	General practice / Family practice
Secondary Subject Heading:	Cardiovascular medicine
Keywords:	PRIMARY CARE, CARDIOLOGY, Anticoagulation < HAEMATOLOGY

SCHOLARONE™
Manuscripts

TITLE

Temporal variation in the diagnosis of resolved atrial fibrillation and the influence of performance targets on clinical coding: cohort study

AUTHORS

Nicola J Adderley[1], Krishnarajah Nirantharakumar[2], Tom Marshall[3]

[1]Research Fellow, Institute of Applied Health Research, University of Birmingham, Edgbaston, Birmingham, B15 2TT

[2]Senior Clinical Lecturer, Institute of Applied Health Research, University of Birmingham, Edgbaston, Birmingham, B15 2TT

[3]Professor of Public Health and Primary Care, Institute of Applied Health Research, University of Birmingham, Edgbaston, Birmingham, B15 2TT

Corresponding author

Tom Marshall

Address: Institute of Applied Health Research, University of Birmingham, Edgbaston, Birmingham, B15 2TT

Email: t.p.marshall@bham.ac.uk

WORD COUNT

Word count (abstract):	294
Word count (main text):	3521
Tables:	0
Figures:	3
References:	18

For peer review only - http://bmjopen.bmj.com/site/about/guidelines.xhtml

ABSTRACT

Objectives

To investigate whether the introduction of performance targets for anticoagulation in atrial fibrillation (AF) was associated with a change in use of the 'resolved atrial fibrillation' code.

Design

Retrospective cohort studies.

Setting

Data from The Health Improvement Network (THIN), a UK database of electronic patient records, from 2000 to 2016.

Participants

250,788 adult patients aged ≥18 years with a diagnosis of AF, including 14,757 with an incident diagnosis of 'resolved AF'.

Main outcome measures

Annual and monthly incidence of 'resolved AF' from 2000 to 2016. Among patients with 'resolved AF', for each year we calculated median duration of the preceding AF diagnosis and the proportion prescribed anticoagulants prior to 'resolved AF'.

Results

Incidence of 'resolved AF' increased from 5.7 to 26.3 per 1000 person-years between 2005 and the introduction of AF performance targets in 2006. Compared to the years prior to the introduction of the performance targets, incidence has remained higher in every year since their implementation. Since 2007, monthly incidence has been highest between January and March. Between 2005 and 2006, median duration between AF and 'resolved AF' diagnoses increased from 276 days (9 months) to 1343 days (3 years 8 months). Among 'resolved AF' patients with CHA_2DS_2-VASc score ≥1, 81.9% (95%CI 81.1 to 82.6) had no current anticoagulant prescription, and 62.3% (95%CI 61.4 to 63.2) had no record of any anticoagulant prescription.

Conclusion

The introduction of AF performance targets was followed by a large increase in use of the 'resolved AF' code, particularly in the months immediately before practices make their anticoagulant performance target submissions. Although most AF patients are prescribed anticoagulants, few patients diagnosed with 'resolved AF' are prescribed anticoagulants and most have never been prescribed them. Untreated patients are much more likely to be coded as having 'resolved AF'.

For peer review only - http://bmjopen.bmj.com/site/about/guidelines.xhtml

Strengths and limitations of this study

- Analysis was performed in a large primary care dataset which is generalisable to the UK population and included more than a quarter of a million patients with atrial fibrillation (AF).
- Data was derived from routinely clinical data which is used by general practitioners for clinical decision-making.
- The study explored the potential impact of the introduction of AF into the Quality and Outcomes Framework on the use of the 'resolved AF' clinical code.
- Use and interpretation of the 'resolved AF' code is likely to vary between general practitioners and practices.
- The primary care dataset contains no direct information on general practitioners' reasons for assigning a 'resolved AF' code; possible influencing factors must therefore be inferred from explorations of temporal variation, patient diagnostic information and anticoagulant prescribing.

For peer review only

For peer review only - http://bmjopen.bmj.com/site/about/guidelines.xhtml

INTRODUCTION

Atrial fibrillation (AF) is a common cardiac arrhythmia associated with increased risk of stroke and transient ischaemic attack (TIA); this increased risk is attenuated by treatment with anticoagulants.[1,2,3] AF may be categorised as resolved if normal heart rhythm is restored. However, AF may recur after apparent resolution. [4,5] Evidence shows that patients diagnosed as having 'resolved AF' continue to be at increased risk of stroke/TIA; from 2013 to 2016, risk in patients with 'resolved AF' was found to be the same as that in patients with ongoing AF.[6]

Factors influencing clinicians to make a diagnosis of 'resolved AF' are unclear. Research has demonstrated that the prevalence of the AF diagnosed clinical code in UK general practice increased significantly after 2006 and has remained comparatively high since.[6] The Quality and Outcomes Framework (QOF) is a scheme to improve the clinical quality of care for chronic diseases. General practices keep a register of patients with particular chronic diseases and are paid an incentive for achieving performance targets for the management of patients on the register. AF was introduced into QOF in 2006 with an incentive payment for ensuring that more than a specified percentage of patients received drugs for stroke prevention.[7] The increase in prevalence of 'resolved AF' after 2006 suggests QOF may have contributed to the increase in 'resolved AF' diagnoses. There was no corresponding jump in the recorded prevalence of AF at this time.[8] From April 2006, general practices were required to maintain a register of patients with AF and to record whether eligible patients were prescribed anticoagulants or antiplatelets. In 2012, the AF QOF indicators were updated to include an assessment of stroke risk and to require patients with a high stroke risk to be treated with anticoagulants (not antiplatelets).[9]

We hypothesised that the introduction of AF into QOF had an impact on the use of the 'resolved AF' code. The aim of this analysis, therefore, was to use information available in routinely collected primary care data to explore this hypothesis by investigating variation in the use of the 'resolved AF' clinical code over time and across different practices, and to investigate other factors which may influence general practitioners to assign a diagnosis of 'resolved AF'. The specific questions addressed were:

1. What is the annual incidence of 'resolved AF' diagnoses and did incidence increase with the introduction of AF into QOF?
2. Since the introduction of AF into QOF, is a diagnosis of 'resolved AF' more likely to be recorded in the months of January to March, immediately prior to the practice QOF submission?
3. Is there a difference in the duration of AF diagnosis in patients diagnosed as having 'resolved AF' before and after the introduction of AF into QOF?
4. Are patients prescribed anticoagulants before their 'resolved AF' diagnosis?
5. How much variation exists between general practices in use of the 'resolved AF' code?

Evidence indicating that use of the 'resolved AF' code may be influenced by QOF reporting would support the recommendation that patients with 'resolved AF' be included in QOF AF registers and receive ongoing AF management,[6] or that the 'resolved AF' clinical code be withdrawn.

For peer review only - http://bmjopen.bmj.com/site/about/guidelines.xhtml

METHODS

Data source

Datasets were extracted from The Health Improvement Network (THIN), a database of electronic primary care records from UK general practices using Vision software. The version of the database from which study datasets were derived included data for approximately 14 million patients at over 640 practices. THIN comprises coded data on patient demographics, diagnoses, prescriptions issued in primary care, consultations and investigations. Data on all prescriptions issued in primary care are recorded in THIN; diagnoses that are part of the QOF are well recorded.

Population

General practices were eligible for participation from the later of the practice acceptable mortality recording (AMR) date,[10] Vision installation date plus one year, and the study start date (1 year prior to the first index/census date).

All adult patients aged 18 years and over with a recorded diagnosis of atrial fibrillation and registered for at least 365 days before the index/census date were eligible for inclusion. AF was defined by a record of a relevant clinical (Read) code.

Study design

A retrospective cohort study from 1st January 2000 to 31st December 2016 was carried out. Index date was the latest of the following two dates: one year after the patient registered with the practice or the date of diagnosis of AF.

To determine incidence of 'resolved AF' among patients with AF, eligible patients were followed up from the index date until the earliest of the following: patient left the practice/transferred out, death, study end date, most recent data upload from practice, or a diagnosis of 'resolved AF'. Patients with a record of 'resolved AF' at study entry were excluded. 'Resolved AF' was defined as a record of the relevant clinical (Read) code (212R.00 'Atrial fibrillation resolved').[6]

To explore temporal variation in AF duration and anticoagulant prescribing preceding a diagnosis of 'resolved AF', a cohort restricted to patients with a diagnosis of 'resolved AF' during the study period was used. Eligible patients were followed up until the earliest of the following: patient left practice/transferred out, death, study end date, most recent data upload from practice, or an outcome event.

To explore practice-level variation in use of the 'resolved AF' clinical code, a cross-sectional study was carried out on 1st December 2016.

For peer review only - http://bmjopen.bmj.com/site/about/guidelines.xhtml

Analysis

Annual incidence of 'resolved AF'

Annual incidence rates of a 'resolved AF' diagnosis among AF patients were calculated for each year from 2000 to 2016 by dividing the number of patients with a new (first) record of 'resolved AF' (numerator) by the total number of person-years at risk (denominator) for the given year.

Monthly variation in use of the 'resolved AF' code pre- and post-QOF

To investigate the impact of QOF on the distribution of 'resolved AF' coding throughout the year, monthly incidence of 'resolved AF' diagnoses (in each month from January to December) was calculated in the pre-QOF period (2000 to 2005), in 2006 and 2007, and in the post-QOF period (2008 to 2016). Monthly incidence was calculated separately for 2006 and 2007 as annual incidence of 'resolved AF' in this period, the years of and immediately following the introduction of AF into QOF, was found to be substantially higher than in subsequent years.

In the post-QOF period (2007 onwards), Poisson regression was used to calculate crude and adjusted incidence rate ratios of stroke/transient ischaemic attack (TIA) in patients with a 'resolved AF' diagnosis recorded in January to March compared to April to December, in order to explore any possible differences in disease severity between patients coded as resolved at different times of the year. The adjusted model included the following covariates: age, sex, CHA_2DS_2-VASc score (categorised as 0, 1 ≥2) and prescription of anticoagulant medication at the time of the 'resolved AF' diagnosis.

'Resolved AF' cohort

The following analyses were restricted to patients with a record of 'resolved AF'.

Duration of AF diagnosis

To explore variation over time in duration of AF diagnosis in patients with 'resolved AF', median (interquartile range, IQR) duration of time between diagnosis of AF (earliest recorded Read code) and first record of a 'resolved AF' code was calculated for each year in patients with a 'resolved AF' code.

Anticoagulant prescribing

To explore prescribing of anticoagulants to patients with a diagnosis of 'resolved AF', the proportion of patients on anticoagulant treatment at the time of diagnosis (current treatment, prescribed up to 90 days prior to 'resolved AF' record), 0 to 90 days, and 91 to 180 days after the 'resolved AF' diagnosis were calculated with 95% CIs for proportions in 1) all 'resolved AF' patients and 2) patients with a CHA_2DS_2-VASc score ≥1 (eligible for anticoagulant treatment). The proportion of 'resolved AF' patients with a CHA_2DS_2-VASc score ≥1 who had never been prescribed anticoagulants was also calculated. Trends over time were explored by calculating the proportions for each year between 2000 and 2016.

For peer review only - http://bmjopen.bmj.com/site/about/guidelines.xhtml

Cross-sectional analysis

Practice-level variation in use of 'resolved AF' code

Variation in use of the 'resolved AF' code by general practice in 2016 was assessed by plotting the percentage of AF patients with any record of a 'resolved AF' code (ever) at a given practice against the number of AF patients at the practice. Upper and lower control limits (within 3 standard deviations of the mean) were calculated.

Definitions of variables

AF, 'resolved AF', and stroke/TIA were defined by the presence of a clinical code; the absence of a clinical code was taken to indicate no diagnosis. The clinical code lists used have been utilised in a number of previous AF studies[6,8,11,12,13] and include all codes used in QOF.[14]

CHA_2DS_2-VASc scores were calculated by adding 1 point each for a history of congestive heart failure (HF), hypertension, diabetes (DM), vascular disease, age 65-74 years and female sex (if another risk factor was present, otherwise 0), and 2 points for age ≥75 and a history of stroke/TIA. HF, hypertension, DM and vascular disease were defined by a relevant clinical code.

Anticoagulants included warfarin, parenteral anticoagulants, other vitamin K antagonists, and novel/non-vitamin K oral anticoagulants.

All statistical analyses were performed in Stata IC version 14.2.

Patient involvement

Patients were not involved in the research.

RESULTS

Annual incidence of 'resolved AF'

A total of 250,788 patients with AF contributing 1,037,858 person-years were included in the analysis; 14,757 patients had an incident diagnosis of 'resolved AF'. Mean (SD) age was 74.6 (12.1) years; 52.6% pf patients were male; median (IQR) follow-up was 3.1 (1.2-6.1) years.

Incidence of the atrial fibrillation (AF) resolved code in patients with AF showed a sharp rise in 2006 (Figure 1), at which time AF was introduced into QOF, rising from 5.7 per 1000 person-years in 2005 to 26.3 per 1000 person-years in 2006. Incidence peaked at 28.6 per 1000 person-years in 2007; it declined thereafter, before rising again to 19.5 per 1000 person-years in 2012-13, when further changes were made to the QOF AF requirements. Since 2013 the incidence has declined.

Monthly variation in use of the 'resolved AF' code

Prior to the introduction of AF into QOF (January 2000 to March 2006), incidence of the 'resolved AF' code remained relatively constant across the 12 months of the year, including the 3 months

For peer review only - http://bmjopen.bmj.com/site/about/guidelines.xhtml

immediately prior to the introduction of AF into QOF (January to March 2006), with monthly incidence varying between 3.2 and 7.2 per 1000 person-years (Figure 2). From April 2006 and for the subsequent 12 months, incidence of the code steadily increased, reaching a peak of 70.2 per 1000 person-years in January 2007. From 2007 onwards (post-QOF), incidence of the 'resolved AF' code has been highest between the months of January and March, the 3 months immediately preceding QOF report submission. In the post-QOF period (2008 to 2016) incidence is higher in every month of the year relative to the same month in the pre-QOF period.

From 2007 onwards, 245 patients diagnosed with 'resolved AF' in January to March and 358 patients diagnosed in April to December had a stroke. Crude incidence rates were 12.4 and 13.8 per 1000 person-years, respectively. Among patients who received a diagnosis of 'resolved AF' after the introduction of AF into QOF (2007 onwards), there was no difference in incidence of stroke/TIA in patients who were assigned the code between January and March compared to those given the code later in the year: crude IRR 0.90 (95% CI 0.76 to 1.06), adjusted IRR 0.98 (95% CI 0.83 to 1.15).

'Resolved AF' cohort

14,863 patients with a record of 'resolved AF' were included in the cohort from 2000 to 2016. Median (IQR) age was 70.7 (59.6-79.6); 58.1% of patients were male. 11,479 (77.2%) patients had a CHA_2DS_2-VASc score ≥1. Median (IQR) follow-up was 3.8 (1.9-6.8) years. 3,384 (22.8%), 1,737 (11.7%) and 9,742 (65.5%) patients had a CHA_2DS_2-VASc score of 0, 1 or ≥2, respectively.

Duration of time between diagnosis of AF and use of the 'resolved AF' code

Median duration of time between diagnosis of AF and first recording of a 'resolved AF' code remained between several months and approximately a year (varying from 69 to 335 days) between 2000 and 2005. In 2006 there was a sharp rise in median duration from 276 days (9 months) in 2005 to 1343 days (3 years 8 months) in 2006. This indicates that in 2006 more than half of patients who were assigned a 'resolved AF' code had been diagnosed over 3 ½ years earlier. Median duration then declined for several years, before rising again to more than 1000 days in 2012-13.

Sequence of events in relation to anticoagulant prescribing in 'resolved AF' patients

Few patients were still on anticoagulants when the 'resolved AF' code was recorded. In the cohort of 'resolved AF' patients (2000 to 2016), 17.3% (95% CI 16.7 to 17.9) had a current prescription at the time of 'resolved AF' recording (up to 90 days prior), with 82.7% (95% CI 82.1 to 83.3) not being prescribed anticoagulant treatment. There was no correlation between anticoagulant prescribing and CHA_2DS_2-VASc category: 14.6%, 25.6% and 16.8% of patients with scores of 0, 1 and ≥2, respectively, were prescribed anticoagulants. This remained true even at high scores: among those with CHA_2DS_2-VASc ≥6, 14.2% were prescribed anticoagulants. Up to 90 days following the 'resolved AF' diagnosis, 9.8% (95% CI 9.3 to 10.3) of patients were still being prescribed anticoagulants. By 91 to 180 days after 'resolved AF', 8.7% (95% CI 8.3 to 9.2) had a prescription for anticoagulants.

Among 'resolved AF' patients with a CHA_2DS_2-VASc score ≥1, 18.1% (95% CI 17.4 to 18.9) had a current prescription for anticoagulants, while 81.9% (95% CI 81.1 to 82.6) had no current prescription. 10.5% (95% CI 10.0 to 11.1) and 9.7% (95% CI 9.2 to 10.3) had prescriptions up to 90

For peer review only - http://bmjopen.bmj.com/site/about/guidelines.xhtml

days and 91 to 180 days following the 'resolved AF' diagnosis respectively. The proportion of 'resolved AF' patients prescribed anticoagulants shortly before and after recording of the 'resolved AF' code varied slightly over time, with a notable drop in 2006 to 9.8% (95% CI 8.5 to 11.4), decreasing from 25.2% (95% CI 20.6 to 30.3) in 2005.

62.3% (95% CI 61.4 to 63.2) of 'resolved AF' patients with a CHA_2DS_2-VASc score ≥1 had no record of an anticoagulant prescription. Among the cohort of patients whose first record of AF was after registration with the practice (n=13,307), 60.6% (95% CI 59.6 to 61.5) had never been prescribed anticoagulants; this proportion varied slightly over time, reaching a peak of 70.2% in 2006 and a low of 51.3% in 2016.

Practice-level variation

787 practices with a total of 1,167,771 patients with AF were included in the analysis from 2000 to 2016. 443 practices with a total of 69,262 patients with AF, of whom 7,261 had a record of 'resolved AF', were included in the analysis in 2016.

Variation in use of the 'resolved AF' code between general practices

The proportion of AF patients with a record of 'resolved AF' varied between practices, ranging from 0% to 43% in 2016. The majority of practices fell within the acceptable range (between the upper (UCL) and lower (LCL) control limits) based on the size of the practice AF population, although a number of practices fell outside this range: 54 (12.2%) practices above the UCL and 30 (6.8%) below the LCL (Figure 3). In 2016, 3 practices with more than 100 patients with AF assigned a 'resolved AF' code to none of these patients, while 10 practices assigned a 'resolved AF' code to more than 25% of patients with AF.

Similar patterns in variation were observed in the year immediately after the introduction of AF into QOF (2007): the proportion of patients with 'resolved AF' ranged from 0% to 40%, with 61 (13.8%) practices above the UCL and 30 (6.8%) below the LCL. In 2005, immediately before the introduction of AF into QOF, there was slightly less variation: the proportion of patients with 'resolved AF' ranged from 0% to 30%, with 39 (8.8%) practices above the UCL. None were below the LCL, which was low due to the smaller average number of patients with 'resolved AF'.

For peer review only

DISCUSSION

Incidence of 'resolved AF' rose dramatically in 2006 immediately following the introduction of AF into the Quality and Outcomes Framework (QOF).[7] Incidence peaked the following year at 28.6 per 1000 person-years, showing a five-fold increase compared to the incidence prior to QOF; it is possible that this increase was in part the result of practices 'catching up' with recording 'resolved AF' following the introduction of QOF. There was a further, smaller, peak in 'resolved AF' incidence in 2012-13, following a change in the QOF AF indicators to introduce a stroke risk assessment indicator and to change the requirements for the anticoagulation indicator.[9] A corresponding rise in the prevalence of 'resolved AF' among patients with AF, from 2.3% in 2005 to 6.4% in 2007 and a high of 9.2% in 2013, has been reported previously.[6]

For peer review only - http://bmjopen.bmj.com/site/about/guidelines.xhtml

Since the introduction of AF into QOF, the majority of 'resolved AF' codes have been recorded between the months of January and March, immediately prior to QOF report submission by general practices. Prior to this, 'resolved AF' codes were recorded throughout the year with little monthly variation in incidence. There is no difference in stroke/TIA rates in patients diagnosed as having 'resolved AF' between January and March compared to those diagnosed later in the year; patients with AF who are diagnosed as resolved immediately prior to QOF do not have a different/lower risk of stroke/TIA.

Immediately following the introduction of AF into QOF, there was a dramatic rise in median duration between AF and 'resolved AF' diagnoses, with a further peak at the time of changes to QOF in 2012-13. At these time points, patients designated as having 'resolved AF' had been diagnosed with AF several years previously (median 3 years and 8 months in 2006) compared around one year prior to QOF (9 months in 2005).

Almost two thirds of patients with 'resolved AF' and a CHA_2DS_2-VASc score ≥1 had never been prescribed anticoagulants. In 2016, 79.5% of patients with 'resolved AF' and a CHA_2DS_2-VASc score ≥1 were not prescribed anticoagulants at the time of their 'resolved AF' diagnosis, made up of 53.5% who had never been prescribed anticoagulants and 26.0% who had previously been prescribed anticoagulants but had subsequently discontinued. By contrast, only 25-30% of patients with ongoing AF and a CHA_2DS_2-VASc score ≥1 were not prescribed anticoagulants in 2016.[8,15] This suggests that patients with AF who are not prescribed anticoagulants may be more likely to be assigned a 'resolved AF' code. Furthermore, recent evidence indicates that patients with a diagnosis of 'resolved AF' remain at increased risk of stroke/TIA and may therefore benefit from continued anticoagulant prophylaxis.[6] The concept of 'resolved AF' may be delusive; AF which has apparently resolved, even following ablation, may recur.[16,17,18]

Use of the 'resolved AF' code varies between practices. Some practices with large numbers of AF patients use the code for very few patients, while others assign the code to more than a quarter of AF patients.

Strengths and limitations

This analysis was performed in a large general practice dataset which is generalisable to the UK population. Data was derived from routinely clinical data which is used by general practitioners for clinical decision-making. The use and interpretation of the 'resolved AF' clinical code is likely to vary between general practitioners and practices. The primary care dataset contains no direct information on general practitioners' reasons for assigning a 'resolved AF' code; possible influencing factors have therefore been inferred from explorations of temporal variation, patient diagnostic information and anticoagulant prescribing. In order to better understand the factors motivating a diagnosis of 'resolved AF', a qualitative study and consultation with practicing clinicians would be required.

Anticoagulation rates may be underestimated if treatment is managed entirely in secondary care; however, the majority of anticoagulants are prescribed in primary care. AF clinical guidelines and stroke risk scoring systems have changed over the study period; for the purpose of this study, we used current guidance (eligibility for anticoagulation based on CHA_2DS_2-VASc score ≥1) across all time periods for consistency and comparability.

For peer review only - http://bmjopen.bmj.com/site/about/guidelines.xhtml

Conclusions

Use of the ‘resolved AF’ code remains common. Most patients eligible for anticoagulant treatment who were assigned a ‘resolved AF’ code were never prescribed anticoagulants, and very few patients were still taking anticoagulants when the ‘resolved AF’ code was recorded. Those diagnosed as having ‘resolved AF’ are no longer included in the AF register for QOF; this has the effect of improving the practice’s apparent performance in the QOF. Incidence of the ‘resolved AF’ clinical code increased markedly when AF was introduced into QOF in 2006 and increased again when further changes were made to the QOF incentive scheme in 2012. Since 2006, incidence of the ‘resolved AF’ code has been highest in the months shortly before practices make their QOF submissions. Previous evidence demonstrated patients with a diagnosis of ‘resolved AF’ remain at increased risk of stroke/TIA and are therefore likely to benefit from anticoagulant prophylaxis. We therefore recommend that patients with ‘resolved AF’ should be included when determining whether practices meet QOF clinical performance targets.

For peer review only

For peer review only - http://bmjopen.bmj.com/site/about/guidelines.xhtml

FIGURE LEGENDS

Figure 1. Annual incidence of resolved atrial fibrillation in patients with AF 2000-2016.

Figure 2. Incidence of the 'resolved AF' code by month of recording, before, during and after the introduction of AF into the Quality and Outcomes Framework (QOF).

Figure 3. Funnel plot showing variation in use of the 'resolved AF' code by practice in 2016.

ETHICAL APPROVAL

The THIN data collection scheme and research carried out using THIN data were approved by the NHS South-East Multicentre Research Ethics Committee (MREC) in 2003; under the terms of this approval, studies must undergo independent scientific review. Approval for these analyses was obtained from the Scientific Review Committee (for the use of THIN data) in April 2015 (15THIN021) and September 2017 (SRC reference number 17THIN082).

COMPETING INTERESTS

All authors have completed the ICMJE uniform disclosure form at http://www.icmje.org/coi_disclosure.pdf and declare: NA and TM report a grant from the National Institute for Health Research (NIHR) Collaboration for Leadership in Applied Health Research and Care West Midlands during the conduct of the study; KN reports funding from AstraZeneca and fees from Sanofi and Boehringer Ingelheim outside the submitted work. Authors declare no other financial relationships with any organisations that might have an interest in the submitted work in the previous three years; and no other relationships or activities that could appear to have influenced the submitted work.

CONTRIBUTORS

NA, KN and TM designed the study. KN undertook data extraction. NA designed and performed the analyses. NA wrote the first draft of the paper, which was revised in collaboration with TM and KN. NA acts as guarantor.

FUNDING

NJA and TM were funded by the NIHR Collaboration for Leadership in Applied Health Research and Care West Midlands initiative (NIHR CLAHRC-WM). This paper presents independent research and the views expressed are those of the author(s) and not necessarily those of the NHS, the NIHR or the Department of Health.

The funders had no role in study design, data collection and analysis, decision to publish, or preparation of the manuscript.

For peer review only

TRANSPARENCY

The lead author (the manuscript's guarantor) affirms that the manuscript is an honest, accurate, and transparent account of the study being reported; that no important aspects of the study have been omitted; and that any discrepancies from the study as planned (and, if relevant, registered) have been explained.

DATA SHARING STATEMENT

Dataset is not available.

EXCLUSIVE LICENCE

I, the Submitting Author has the right to grant and does grant on behalf of all authors of the Work (as defined in the below author licence), an exclusive licence and/or a non-exclusive licence for contributions from authors who are: i) UK Crown employees; ii) where BMJ has agreed a CC-BY licence shall apply, and/or iii) in accordance with the terms applicable for US Federal Government officers or employees acting as part of their official duties; on a worldwide, perpetual, irrevocable, royalty-free basis to BMJ Publishing Group Ltd ("BMJ") its licensees and where the relevant Journal is co-owned by BMJ to the co-owners of the Journal, to publish the Work in BMJ Open and any other BMJ products and to exploit all rights, as set out in our licence.

For peer review only

For peer review only - http://bmjopen.bmj.com/site/about/guidelines.xhtml

REFERENCES

[1] Antithrombotic Trialists' Collaboration. Collaborative meta-analysis of randomised trials of antiplatelet therapy for prevention of death, myocardial infarction, and stroke in high risk patients. *BMJ* 2002; 324:71–86.

[2] Aguilar MI and Hart R. Oral anticoagulants for preventing stroke in patients with non-valvular atrial fibrillation and no previous history of stroke or transient ischemic attacks. Cochrane Database of Systematic Reviews 2005, Issue 3. Art. No.: CD001927. doi: 10.1002/14651858.CD001927.pub2.

[3] Aguilar MI, Hart R and Pearce LA. Oral anticoagulants versus antiplatelet therapy for preventing stroke in patients with non-valvular atrial fibrillation and no history of stroke or transient ischemic attacks. *Cochrane Database of Systematic Reviews* 2007, Issue 3. Art. No.: CD006186. doi: 10.1002/14651858.CD006186.pub2.

[4] Wynn GJ, El-Kadri M, Haq I, Das M, Modi S, Snowdon R, Hall M, Waktare JE, Todd DM, Gupta D. Long-term outcomes after ablation of persistent atrial fibrillation: an observational study over 6 years. Open Heart. 2016;3:e000394. doi: 10.1136/openhrt-2015-000394

[5] Militaru C, Donoiu I. Atrial Fibrillation Recurrence Predictors after Conversion to Sinus Rhythm Curr Health Sci J. 2014 Apr-Jun; 40(2): 129–133. doi:10.12865/CHSJ.40.02.09

[6] Adderley NJ, NIrantharakumar K, Marshall T. Risk of stroke and transient ischaemic attack in patients with a diagnosis of resolved atrial fibrillation: retrospective cohort studies. BMJ 2018. doi:10.1136/bmj.k1717

[7] NHS Employers and General Practitioners Committee. Revisions to the GMS contract 2006/07. Delivering investment in general practice. NHS Employers: London 2006.

[8] Adderley NJ, Ryan R, Nirantharakumar K, Marshall T. Prevalence and treatment of atrial fibrillation in UK general practice from 2000 to 2016. Heart 2018. doi:10.1136/heartjnl-2018-312977

[9] NHS Employers and General Practitioners Committee. Quality and Outcomes Framework for 2012/13. Guidance for PCOs and practices. NHS Employers: London 2012.

[10] Maguire A, Blak BT, Thompson M. The importance of defining periods of complete mortality reporting for research using automated data from primary care. *Pharmacoepidemiol Drug Saf* 2009;18:76e83.

[11] Cowan C, Healicon R, Robson I, et al. The use of anticoagulants in the management of atrial fibrillation among general practices in England. *Heart* 2013;99:1166–72. doi: 10.1136/heartjnl-2012-303472

[12] Adderley N, Ryan R, Marshall T. The role of contraindications in prescribing anticoagulants to patients with atrial fibrillation: a cross-sectional analysis of primary care data in the UK. *Br J Gen Prac* 2017. doi: 10.3399/bjgp17X691685

[13] Isaew A, Adderley NJ, Ryan R, Fitzmaurice D, Marshall T. The treatment of paroxysmal atrial fibrillation in UK primary care. *Heart* 2017;103:1502-7. doi: 10.1136/heartjnl-2016-310927

[14] NHS England. New GMS Contract QOF Implementation. Dataset and Business Rules. Atrial Fibrillation Indicator Set. Leeds: Health and Social Care Information Centre 2016.

[15] Lacoin L, Lumley M, Ridha E, Pereira M, McDonald L, Ramagopalan S, et al. Evolving landscape of stroke prevention in atrial fibrillation within the UK between 2012 and 2016: a cross-sectional analysis study using CPRD. BMJ Open 2017;7:e015363. doi:10.1136/bmjopen-2016-015363

[16] Chao TF, Lin YJ, Chang SL, et al. Can oral anticoagulants be stopped safely after a successful atrial fibrillation ablation? *J Thorac Dis* 2015;7:172-7. doi: 10.3978/j.issn.2072-1439.2015.01.18

For peer review only - http://bmjopen.bmj.com/site/about/guidelines.xhtml

[17] Chao TF, Tsao HM, Lin YJ, et al. Clinical outcome of catheter ablation in patients with nonparoxysmal atrial fibrillation: results of 3-year follow-up. *Circ Arrhythm Electrophysiol* 2012;5:514-20.

[18] Tilz RR, Rillig A, Thum AM, et al. Catheter ablation of long-standing persistent atrial fibrillation: 5-year outcomes of the Hamburg Sequential Ablation Strategy. *J Am Coll Cardiol* 2012;60:1921-9.

For peer review only

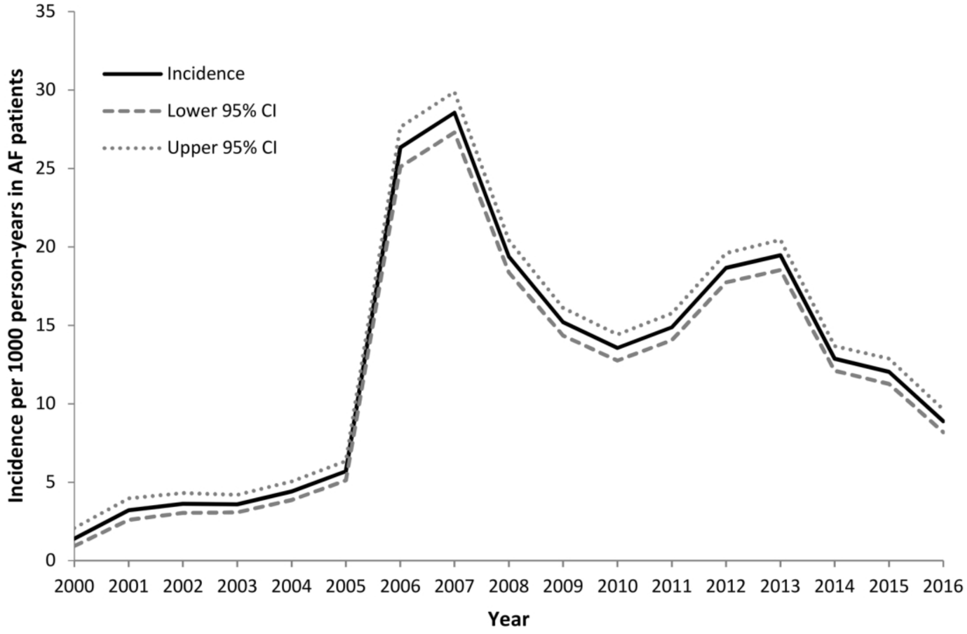

Figure 1. Annual incidence of resolved atrial fibrillation in patients with AF 2000-2016.

157x105mm (300 x 300 DPI)

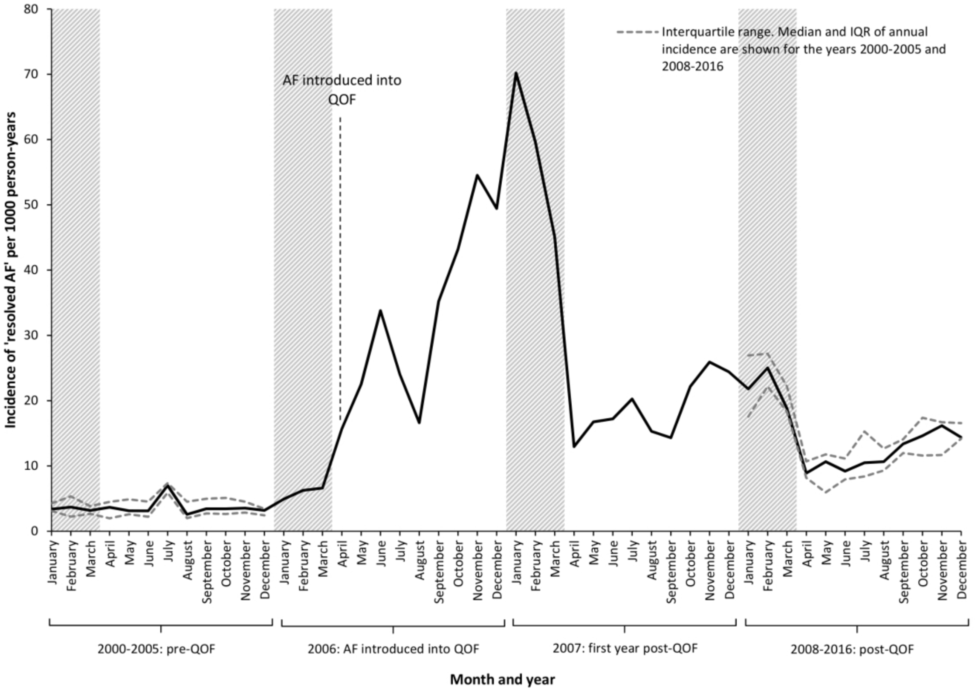

Figure 2. Incidence of the 'resolved AF' code by month of recording, before, during and after the introduction of AF into the Quality and Outcomes Framework (QOF).

223x157mm (299 x 299 DPI)

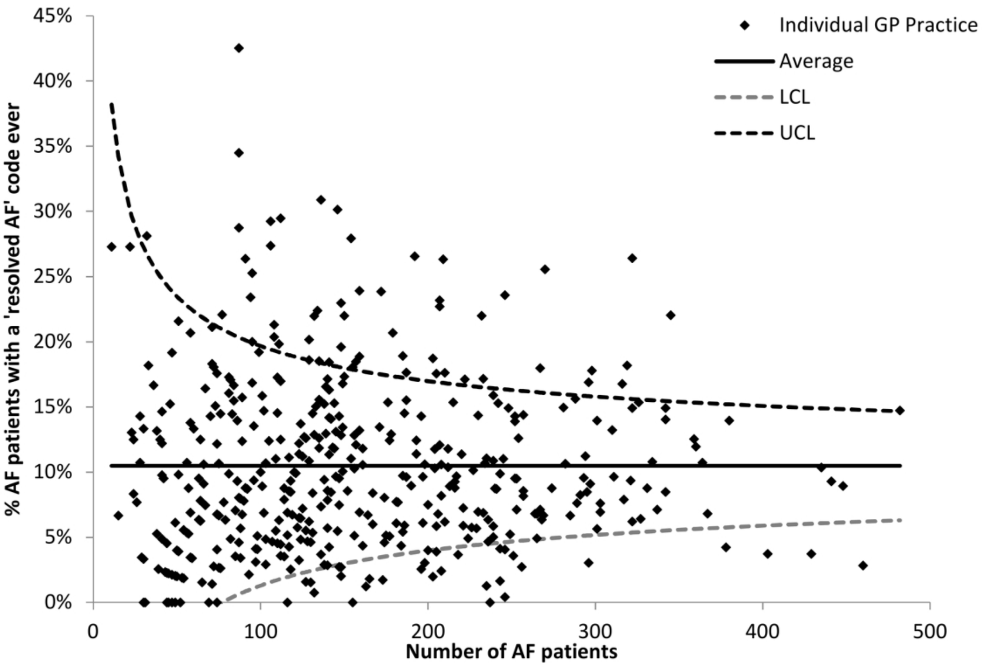

Figure 3. Funnel plot showing variation in use of the 'resolved AF' code by practice in 2016.

155x104mm (300 x 300 DPI)

The RECORD statement – checklist of items, extended from the STROBE statement, that should be reported in observational studies using routinely collected health data.

	Item No.	STROBE items	Location in manuscript where items are reported	RECORD items	Location in manuscript where items are reported
**Title and abstract**					
	1	(a) Indicate the study's design with a commonly used term in the title or the abstract (b) Provide in the abstract an informative and balanced summary of what was done and what was found	p.3	RECORD 1.1: The type of data used should be specified in the title or abstract. When possible, the name of the databases used should be included.<br><br>RECORD 1.2: If applicable, the geographic region and timeframe within which the study took place should be reported in the title or abstract.<br><br>RECORD 1.3: If linkage between databases was conducted for the study, this should be clearly stated in the title or abstract.	p.3<br><br>p.3<br><br>n/a
**Introduction**					
Background rationale	2	Explain the scientific background and rationale for the investigation being reported	p.4		
Objectives	3	State specific objectives, including any prespecified hypotheses	p.4		
**Methods**					
Study Design	4	Present key elements of study design early in the paper	pp.2, 4-6		
Setting	5	Describe the setting, locations, and relevant dates, including periods of recruitment, exposure, follow-up, and data collection	pp.4-5		

For peer review only

Participants	6	*(a) Cohort study* - Give the eligibility criteria, and the sources and methods of selection of participants. Describe methods of follow-up<br>*Case-control study* - Give the eligibility criteria, and the sources and methods of case ascertainment and control selection. Give the rationale for the choice of cases and controls<br>*Cross-sectional study* - Give the eligibility criteria, and the sources and methods of selection of participants<br><br>*(b) Cohort study* - For matched studies, give matching criteria and number of exposed and unexposed<br>*Case-control study* - For matched studies, give matching criteria and the number of controls per case	pp.5-6<br>n/a<br>pp.5-6<br>n/a<br>n/a	RECORD 6.1: The methods of study population selection (such as codes or algorithms used to identify subjects) should be listed in detail. If this is not possible, an explanation should be provided.<br><br>RECORD 6.2: Any validation studies of the codes or algorithms used to select the population should be referenced. If validation was conducted for this study and not published elsewhere, detailed methods and results should be provided.<br><br>RECORD 6.3: If the study involved linkage of databases, consider use of a flow diagram or other graphical display to demonstrate the data linkage process, including the number of individuals with linked data at each stage.	pp.5-6<br>p.5<br>n/a
Variables	7	Clearly define all outcomes, exposures, predictors, potential confounders, and effect modifiers. Give diagnostic criteria, if applicable.	pp.5-6	RECORD 7.1: A complete list of codes and algorithms used to classify exposures, outcomes, confounders, and effect modifiers should be provided. If these cannot be reported, an explanation should be provided.	pp.5-6
Data sources/ measurement	8	For each variable of interest, give sources of data and details of methods of assessment (measurement).<br>Describe comparability of assessment methods if there is more than one group	pp.5-6		

For peer review only

Bias	9	Describe any efforts to address potential sources of bias	pp.9-10		
Study size	10	Explain how the study size was arrived at	n/a – full sample		
Quantitative variables	11	Explain how quantitative variables were handled in the analyses. If applicable, describe which groupings were chosen, and why	pp.5-6		
Statistical methods	12	(a) Describe all statistical methods, including those used to control for confounding<br>(b) Describe any methods used to examine subgroups and interactions<br>(c) Explain how missing data were addressed<br>(d) *Cohort study* - If applicable, explain how loss to follow-up was addressed<br>*Case-control study* - If applicable, explain how matching of cases and controls was addressed<br>*Cross-sectional study* - If applicable, describe analytical methods taking account of sampling strategy<br>(e) Describe any sensitivity analyses	pp.5-6<br>n/a<br>n/a<br>n/a<br>n/a<br>n/a<br>n/a		
Data access and cleaning methods		..		RECORD 12.1: Authors should describe the extent to which the investigators had access to the database population used to create the study population.	p.4

For peer review only

				RECORD 12.2: Authors should provide information on the data cleaning methods used in the study.	p.5
Linkage		..		RECORD 12.3: State whether the study included person-level, institutional-level, or other data linkage across two or more databases. The methods of linkage and methods of linkage quality evaluation should be provided.	n/a
**Results**					
Participants	13	(a) Report the numbers of individuals at each stage of the study (*e.g.*, numbers potentially eligible, examined for eligibility, confirmed eligible, included in the study, completing follow-up, and analysed)<br>(b) Give reasons for non-participation at each stage.<br>(c) Consider use of a flow diagram	pp.7-8<br><br>n/a<br><br>n/a	RECORD 13.1: Describe in detail the selection of the persons included in the study (*i.e.,* study population selection) including filtering based on data quality, data availability and linkage. The selection of included persons can be described in the text and/or by means of the study flow diagram.	pp.5-7
Descriptive data	14	(a) Give characteristics of study participants (*e.g.*, demographic, clinical, social) and information on exposures and potential confounders<br>(b) Indicate the number of participants with missing data for each variable of interest<br>(c) *Cohort study* - summarise follow-up time (*e.g.*, average and total amount)	pp.7-8<br><br>n/a<br><br>pp.7-8		
Outcome data	15	*Cohort study* - Report numbers of outcome events or summary measures over time	p.7		

For peer review only

		Case-control study - Report numbers in each exposure category, or summary measures of exposure<br>*Cross-sectional study* - Report numbers of outcome events or summary measures	n/a<br><br><br><br>p.8		
Main results	16	(a) Give unadjusted estimates and, if applicable, confounder-adjusted estimates and their precision (e.g., 95% confidence interval). Make clear which confounders were adjusted for and why they were included<br>(b) Report category boundaries when continuous variables were categorized<br>(c) If relevant, consider translating estimates of relative risk into absolute risk for a meaningful time period	pp.7-8<br><br><br><br><br><br><br>n/a<br><br><br>n/a		
Other analyses	17	Report other analyses done—e.g., analyses of subgroups and interactions, and sensitivity analyses	n/a		
**Discussion**					
Key results	18	Summarise key results with reference to study objectives	p.9		
Limitations	19	Discuss limitations of the study, taking into account sources of potential bias or imprecision. Discuss both direction and magnitude of any potential bias	pp.9-10	RECORD 19.1: Discuss the implications of using data that were not created or collected to answer the specific research question(s). Include discussion of misclassification bias, unmeasured confounding, missing data, and changing eligibility over time, as they pertain to the study being reported.	pp.9-10

For peer review only

Interpretation	20	Give a cautious overall interpretation of results considering objectives, limitations, multiplicity of analyses, results from similar studies, and other relevant evidence	p.10		
Generalisability	21	Discuss the generalisability (external validity) of the study results	p.9		
**Other Information**					
Funding	22	Give the source of funding and the role of the funders for the present study and, if applicable, for the original study on which the present article is based	p.11		
Accessibility of protocol, raw data, and programming code		..		RECORD 22.1: Authors should provide information on how to access any supplemental information such as the study protocol, raw data, or programming code.	n/a

*Reference: Benchimol EI, Smeeth L, Guttmann A, Harron K, Moher D, Petersen I, Sørensen HT, von Elm E, Langan SM, the RECORD Working Committee. The REporting of studies Conducted using Observational Routinely-collected health Data (RECORD) Statement. *PLoS Medicine* 2015; in press.

*Checklist is protected under Creative Commons Attribution (CC BY) license.

For peer review only

BMJ Open

Temporal variation in the diagnosis of resolved atrial fibrillation and the influence of performance targets on clinical coding: cohort study

Journal:	*BMJ Open*
Manuscript ID	bmjopen-2019-030454.R3
Article Type:	Original research
Date Submitted by the Author:	16-Oct-2019
Complete List of Authors:	Adderley, Nicola; University of Birmingham, Institute of Applied Health Research<br>Nirantharakumar, Krishnarajah; University of Birmingham, Institute of Applied Health Research<br>Marshall, Tom; University of Birmingham, Institute of Applied Health Research
**Primary Subject Heading**:	General practice / Family practice
Secondary Subject Heading:	Cardiovascular medicine
Keywords:	PRIMARY CARE, CARDIOLOGY, Anticoagulation < HAEMATOLOGY

SCHOLARONE™
Manuscripts

TITLE

Temporal variation in the diagnosis of resolved atrial fibrillation and the influence of performance targets on clinical coding: cohort study

AUTHORS

Nicola J Adderley[1], Krishnarajah Nirantharakumar[2], Tom Marshall[3]

[1]Research Fellow, Institute of Applied Health Research, University of Birmingham, Edgbaston, Birmingham, B15 2TT

[2]Senior Clinical Lecturer, Institute of Applied Health Research, University of Birmingham, Edgbaston, Birmingham, B15 2TT

[3]Professor of Public Health and Primary Care, Institute of Applied Health Research, University of Birmingham, Edgbaston, Birmingham, B15 2TT

Corresponding author

Tom Marshall

Address: Institute of Applied Health Research, University of Birmingham, Edgbaston, Birmingham, B15 2TT

Email: t.p.marshall@bham.ac.uk

WORD COUNT

Word count (abstract):	294
Word count (main text):	3533
Tables:	0
Figures:	3
References:	18

For peer review only

For peer review only - http://bmjopen.bmj.com/site/about/guidelines.xhtml

ABSTRACT

Objectives

To investigate whether the introduction of performance targets for anticoagulation in atrial fibrillation (AF) was associated with a change in use of the 'resolved atrial fibrillation' code.

Design

Retrospective cohort studies.

Setting

Data from The Health Improvement Network (THIN), a UK database of electronic patient records, from 2000 to 2016.

Participants

250,788 adult patients aged ≥18 years with a diagnosis of AF, including 14,757 with an incident diagnosis of 'resolved AF'.

Main outcome measures

Annual and monthly incidence of 'resolved AF' from 2000 to 2016. Among patients with 'resolved AF', for each year we calculated median duration of the preceding AF diagnosis and the proportion prescribed anticoagulants prior to 'resolved AF'.

Results

Incidence of 'resolved AF' increased from 5.7 to 26.3 per 1000 person-years between 2005 and the introduction of AF performance targets in 2006. Compared to the years prior to the introduction of the performance targets, incidence has remained higher in every year since their implementation. Since 2007, monthly incidence has been highest between January and March. Between 2005 and 2006, median duration between AF and 'resolved AF' diagnoses increased from 276 days (9 months) to 1343 days (3 years 8 months). Among 'resolved AF' patients with CHA_2DS_2-VASc score ≥1, 81.9% (95%CI 81.1 to 82.6) had no current anticoagulant prescription, and 62.3% (95%CI 61.4 to 63.2) had no record of any anticoagulant prescription.

Conclusion

The introduction of AF performance targets was followed by a large increase in use of the 'resolved AF' code, particularly in the months immediately before practices make their anticoagulant performance target submissions. Although most AF patients are prescribed anticoagulants, few patients diagnosed with 'resolved AF' are prescribed anticoagulants and most have never been prescribed them. Untreated patients are much more likely to be coded as having 'resolved AF'.

For peer review only - http://bmjopen.bmj.com/site/about/guidelines.xhtml

Strengths and limitations of this study

- Analysis was performed in a large primary care dataset which is generalisable to the UK population and included more than a quarter of a million patients with atrial fibrillation (AF).
- Data was derived from routinely clinical data which is used by general practitioners for clinical decision-making.
- The study explored the potential impact of the introduction of AF into the Quality and Outcomes Framework on the use of the 'resolved AF' clinical code.
- Use and interpretation of the 'resolved AF' code is likely to vary between general practitioners and practices.
- The primary care dataset contains no direct information on general practitioners' reasons for assigning a 'resolved AF' code; possible influencing factors must therefore be inferred from explorations of temporal variation, patient diagnostic information and anticoagulant prescribing.

For peer review only

For peer review only - http://bmjopen.bmj.com/site/about/guidelines.xhtml

INTRODUCTION

Atrial fibrillation (AF) is a common cardiac arrhythmia associated with increased risk of stroke and transient ischaemic attack (TIA); this increased risk is attenuated by treatment with anticoagulants.[1,2,3] AF may be categorised as resolved if normal heart rhythm is restored. However, AF may recur after apparent resolution. [4,5] Evidence shows that patients diagnosed as having 'resolved AF' continue to be at increased risk of stroke/TIA; from 2013 to 2016, risk in patients with 'resolved AF' was found to be the same as that in patients with ongoing AF.[6]

Factors influencing clinicians to make a diagnosis of 'resolved AF' are unclear. Research has demonstrated that the prevalence of the AF resolved clinical code in UK general practice increased significantly after 2006 and has remained comparatively high since.[6] The Quality and Outcomes Framework (QOF) is a scheme to improve the clinical quality of care for chronic diseases. General practices keep a register of patients with particular chronic diseases and are paid an incentive for achieving performance targets for the management of patients on the register. AF was introduced into QOF in 2006 with an incentive payment for ensuring that more than a specified percentage of patients received drugs for stroke prevention.[7] From April 2006, general practices were required to maintain a register of patients with AF and to record whether eligible patients were prescribed anticoagulants or antiplatelets; patients with a code indicating 'resolved AF' are excluded from this register. The increase in prevalence of 'resolved AF' after 2006 suggests QOF may have contributed to the increase in 'resolved AF' diagnoses. There was no corresponding jump in the recorded prevalence of AF at this time.[8] In 2012, the AF QOF indicators were updated to include an assessment of stroke risk and to require patients with a high stroke risk to be treated with anticoagulants (not antiplatelets).[9]

We hypothesised that the introduction of AF into QOF had an impact on the use of the 'resolved AF' code. The aim of this analysis, therefore, was to use information available in routinely collected primary care data to explore this hypothesis by investigating variation in the use of the 'resolved AF' clinical code over time and across different practices, and to investigate other factors which may influence general practitioners to assign a diagnosis of 'resolved AF'. The specific questions addressed were:

1. What is the annual incidence of 'resolved AF' diagnoses and did incidence increase with the introduction of AF into QOF?
2. Since the introduction of AF into QOF, is a diagnosis of 'resolved AF' more likely to be recorded in the months of January to March, immediately prior to the practice QOF submission?
3. Is there a difference in the duration of AF diagnosis in patients diagnosed as having 'resolved AF' before and after the introduction of AF into QOF?
4. Are patients prescribed anticoagulants before their 'resolved AF' diagnosis?
5. How much variation exists between general practices in use of the 'resolved AF' code?

Evidence indicating that use of the 'resolved AF' code may be influenced by QOF reporting would support the recommendation that patients with 'resolved AF' be included in QOF AF registers and receive ongoing AF management,[6] or that the 'resolved AF' clinical code be withdrawn.

For peer review only - http://bmjopen.bmj.com/site/about/guidelines.xhtml

METHODS

Data source

Datasets were extracted from The Health Improvement Network (THIN), a database of electronic primary care records from UK general practices using Vision software. The version of the database from which study datasets were derived included data for approximately 14 million patients at over 640 practices. THIN comprises coded data on patient demographics, diagnoses, prescriptions issued in primary care, consultations and investigations. Data on all prescriptions issued in primary care are recorded in THIN; diagnoses that are part of the QOF are well recorded.

Population

General practices were eligible for participation from the later of the practice acceptable mortality recording (AMR) date,[10] Vision installation date plus one year, and the study start date (1 year prior to the first index/census date).

All adult patients aged 18 years and over with a recorded diagnosis of atrial fibrillation and registered for at least 365 days before the index/census date were eligible for inclusion. AF was defined by a record of a relevant clinical (Read) code.

Study design

A retrospective cohort study from 1st January 2000 to 31st December 2016 was carried out. Index date was the latest of the following two dates: one year after the patient registered with the practice or the date of diagnosis of AF.

To determine incidence of 'resolved AF' among patients with AF, eligible patients were followed up from the index date until the earliest of the following: patient left the practice/transferred out, death, study end date, most recent data upload from practice, or a diagnosis of 'resolved AF'. Patients with a record of 'resolved AF' at study entry were excluded. 'Resolved AF' was defined as a record of the relevant clinical (Read) code (212R.00 'Atrial fibrillation resolved').[6]

To explore temporal variation in AF duration and anticoagulant prescribing preceding a diagnosis of 'resolved AF', a cohort restricted to patients with a diagnosis of 'resolved AF' during the study period was used. Eligible patients were followed up until the earliest of the following: patient left practice/transferred out, death, study end date, most recent data upload from practice, or an outcome event.

To explore practice-level variation in use of the 'resolved AF' clinical code, a cross-sectional study was carried out on 1st December 2016.

For peer review only - http://bmjopen.bmj.com/site/about/guidelines.xhtml

Analysis

Annual incidence of 'resolved AF'

Annual incidence rates of a 'resolved AF' diagnosis among AF patients were calculated for each year from 2000 to 2016 by dividing the number of patients with a new (first) record of 'resolved AF' (numerator) by the total number of person-years at risk (denominator) for the given year.

Monthly variation in use of the 'resolved AF' code pre- and post-QOF

To investigate the impact of QOF on the distribution of 'resolved AF' coding throughout the year, monthly incidence of 'resolved AF' diagnoses (in each month from January to December) was calculated in the pre-QOF period (2000 to 2005), in 2006 and 2007, and in the post-QOF period (2008 to 2016). Monthly incidence was calculated separately for 2006 and 2007 as annual incidence of 'resolved AF' in this period, the years of and immediately following the introduction of AF into QOF, was found to be substantially higher than in subsequent years.

In the post-QOF period (2007 onwards), Poisson regression was used to calculate crude and adjusted incidence rate ratios of stroke/transient ischaemic attack (TIA) in patients with a 'resolved AF' diagnosis recorded in January to March compared to April to December, in order to explore any possible differences in disease severity between patients coded as resolved at different times of the year. The adjusted model included the following covariates: age, sex, CHA_2DS_2-VASc score (categorised as 0, 1 ≥2) and prescription of anticoagulant medication at the time of the 'resolved AF' diagnosis.

'Resolved AF' cohort

The following analyses were restricted to patients with a record of 'resolved AF'.

Duration of AF diagnosis

To explore variation over time in duration of AF diagnosis in patients with 'resolved AF', median (interquartile range, IQR) duration of time between diagnosis of AF (earliest recorded Read code) and first record of a 'resolved AF' code was calculated for each year in patients with a 'resolved AF' code.

Anticoagulant prescribing

To explore prescribing of anticoagulants to patients with a diagnosis of 'resolved AF', the proportion of patients on anticoagulant treatment at the time of diagnosis (current treatment, prescribed up to 90 days prior to 'resolved AF' record), 0 to 90 days, and 91 to 180 days after the 'resolved AF' diagnosis were calculated with 95% CIs for proportions in 1) all 'resolved AF' patients and 2) patients with a CHA_2DS_2-VASc score ≥1 (eligible for anticoagulant treatment). The proportion of 'resolved AF' patients with a CHA_2DS_2-VASc score ≥1 who had never been prescribed anticoagulants was also calculated. Trends over time were explored by calculating the proportions for each year between 2000 and 2016.

For peer review only - http://bmjopen.bmj.com/site/about/guidelines.xhtml

Cross-sectional analysis

Practice-level variation in use of 'resolved AF' code

Variation in use of the 'resolved AF' code by general practice in 2016 was assessed by plotting the percentage of AF patients with any record of a 'resolved AF' code (ever) at a given practice against the number of AF patients at the practice. Upper and lower control limits (within 3 standard deviations of the mean) were calculated.

Definitions of variables

AF, 'resolved AF', and stroke/TIA were defined by the presence of a clinical code; the absence of a clinical code was taken to indicate no diagnosis. The clinical code lists used have been utilised in a number of previous AF studies[6,8,11,12,13] and include all codes used in QOF.[14]

CHA_2DS_2-VASc scores were calculated by adding 1 point each for a history of congestive heart failure (HF), hypertension, diabetes (DM), vascular disease, age 65-74 years and female sex (if another risk factor was present, otherwise 0), and 2 points for age ≥75 and a history of stroke/TIA. HF, hypertension, DM and vascular disease were defined by a relevant clinical code.

Anticoagulants included warfarin, parenteral anticoagulants, other vitamin K antagonists, and novel/non-vitamin K oral anticoagulants.

All statistical analyses were performed in Stata IC version 14.2.

Patient involvement

Patients were not involved in the research.

RESULTS

Annual incidence of 'resolved AF'

A total of 250,788 patients with AF contributing 1,037,858 person-years were included in the analysis; 14,757 patients had an incident diagnosis of 'resolved AF'. Mean (SD) age was 74.6 (12.1) years; 52.6% pf patients were male; median (IQR) follow-up was 3.1 (1.2-6.1) years.

Incidence of the atrial fibrillation (AF) resolved code in patients with AF showed a sharp rise in 2006 (Figure 1), at which time AF was introduced into QOF, rising from 5.7 per 1000 person-years in 2005 to 26.3 per 1000 person-years in 2006. Incidence peaked at 28.6 per 1000 person-years in 2007; it declined thereafter, before rising again to 19.5 per 1000 person-years in 2012-13, when further changes were made to the QOF AF requirements. Since 2013 the incidence has declined.

Monthly variation in use of the 'resolved AF' code

Prior to the introduction of AF into QOF (January 2000 to March 2006), incidence of the 'resolved AF' code remained relatively constant across the 12 months of the year, including the 3 months

For peer review only - http://bmjopen.bmj.com/site/about/guidelines.xhtml

For peer review only

immediately prior to the introduction of AF into QOF (January to March 2006), with monthly incidence varying between 3.2 and 7.2 per 1000 person-years (Figure 2). From April 2006 and for the subsequent 12 months, incidence of the code steadily increased, reaching a peak of 70.2 per 1000 person-years in January 2007. From 2007 onwards (post-QOF), incidence of the 'resolved AF' code has been highest between the months of January and March, the 3 months immediately preceding QOF report submission. In the post-QOF period (2008 to 2016) incidence is higher in every month of the year relative to the same month in the pre-QOF period.

From 2007 onwards, 245 patients diagnosed with 'resolved AF' in January to March and 358 patients diagnosed in April to December had a stroke. Crude incidence rates were 12.4 and 13.8 per 1000 person-years, respectively. Among patients who received a diagnosis of 'resolved AF' after the introduction of AF into QOF (2007 onwards), there was no difference in incidence of stroke/TIA in patients who were assigned the code between January and March compared to those given the code later in the year: crude IRR 0.90 (95% CI 0.76 to 1.06), adjusted IRR 0.98 (95% CI 0.83 to 1.15).

'Resolved AF' cohort

14,863 patients with a record of 'resolved AF' were included in the cohort from 2000 to 2016. Median (IQR) age was 70.7 (59.6-79.6); 58.1% of patients were male. 11,479 (77.2%) patients had a CHA_2DS_2-VASc score ≥1. Median (IQR) follow-up was 3.8 (1.9-6.8) years. 3,384 (22.8%), 1,737 (11.7%) and 9,742 (65.5%) patients had a CHA_2DS_2-VASc score of 0, 1 or ≥2, respectively.

Duration of time between diagnosis of AF and use of the 'resolved AF' code

Median duration of time between diagnosis of AF and first recording of a 'resolved AF' code remained between several months and approximately a year (varying from 69 to 335 days) between 2000 and 2005. In 2006 there was a sharp rise in median duration from 276 days (9 months) in 2005 to 1343 days (3 years 8 months) in 2006. This indicates that in 2006 more than half of patients who were assigned a 'resolved AF' code had been diagnosed over 3 ½ years earlier. Median duration then declined for several years, before rising again to more than 1000 days in 2012-13.

Sequence of events in relation to anticoagulant prescribing in 'resolved AF' patients

Few patients were still on anticoagulants when the 'resolved AF' code was recorded. In the cohort of 'resolved AF' patients (2000 to 2016), 17.3% (95% CI 16.7 to 17.9) had a current prescription at the time of 'resolved AF' recording (up to 90 days prior), with 82.7% (95% CI 82.1 to 83.3) not being prescribed anticoagulant treatment. There was no correlation between anticoagulant prescribing and CHA_2DS_2-VASc category: 14.6%, 25.6% and 16.8% of patients with scores of 0, 1 and ≥2, respectively, were prescribed anticoagulants. This remained true even at high scores: among those with CHA_2DS_2-VASc ≥6, 14.2% were prescribed anticoagulants. Up to 90 days following the 'resolved AF' diagnosis, 9.8% (95% CI 9.3 to 10.3) of patients were still being prescribed anticoagulants. By 91 to 180 days after 'resolved AF', 8.7% (95% CI 8.3 to 9.2) had a prescription for anticoagulants.

Among 'resolved AF' patients with a CHA_2DS_2-VASc score ≥1, 18.1% (95% CI 17.4 to 18.9) had a current prescription for anticoagulants, while 81.9% (95% CI 81.1 to 82.6) had no current prescription. 10.5% (95% CI 10.0 to 11.1) and 9.7% (95% CI 9.2 to 10.3) had prescriptions up to 90

For peer review only - http://bmjopen.bmj.com/site/about/guidelines.xhtml

For peer review only

days and 91 to 180 days following the 'resolved AF' diagnosis respectively. The proportion of 'resolved AF' patients prescribed anticoagulants shortly before and after recording of the 'resolved AF' code varied slightly over time, with a notable drop in 2006 to 9.8% (95% CI 8.5 to 11.4), decreasing from 25.2% (95% CI 20.6 to 30.3) in 2005.

62.3% (95% CI 61.4 to 63.2) of 'resolved AF' patients with a CHA_2DS_2-VASc score ≥1 had no record of an anticoagulant prescription. Among the cohort of patients whose first record of AF was after registration with the practice (n=13,307), 60.6% (95% CI 59.6 to 61.5) had never been prescribed anticoagulants; this proportion varied slightly over time, reaching a peak of 70.2% in 2006 and a low of 51.3% in 2016.

Practice-level variation

787 practices with a total of 1,167,771 patients with AF were included in the analysis from 2000 to 2016. 443 practices with a total of 69,262 patients with AF, of whom 7,261 had a record of 'resolved AF', were included in the analysis in 2016.

Variation in use of the 'resolved AF' code between general practices

The proportion of AF patients with a record of 'resolved AF' varied between practices, ranging from 0% to 43% in 2016. The majority of practices fell within the acceptable range (between the upper (UCL) and lower (LCL) control limits) based on the size of the practice AF population, although a number of practices fell outside this range: 54 (12.2%) practices above the UCL and 30 (6.8%) below the LCL (Figure 3). In 2016, 3 practices with more than 100 patients with AF assigned a 'resolved AF' code to none of these patients, while 10 practices assigned a 'resolved AF' code to more than 25% of patients with AF.

Similar patterns in variation were observed in the year immediately after the introduction of AF into QOF (2007): the proportion of patients with 'resolved AF' ranged from 0% to 40%, with 61 (13.8%) practices above the UCL and 30 (6.8%) below the LCL. In 2005, immediately before the introduction of AF into QOF, there was slightly less variation: the proportion of patients with 'resolved AF' ranged from 0% to 30%, with 39 (8.8%) practices above the UCL. None were below the LCL, which was low due to the smaller average number of patients with 'resolved AF'.

For peer review only

DISCUSSION

Incidence of 'resolved AF' rose dramatically in 2006 immediately following the introduction of AF into the Quality and Outcomes Framework (QOF).[7] Incidence peaked the following year at 28.6 per 1000 person-years, showing a five-fold increase compared to the incidence prior to QOF; it is possible that this increase was in part the result of practices 'catching up' with recording 'resolved AF' following the introduction of QOF. There was a further, smaller, peak in 'resolved AF' incidence in 2012-13, following a change in the QOF AF indicators to introduce a stroke risk assessment indicator and to change the requirements for the anticoagulation indicator.[9] A corresponding rise in the prevalence of 'resolved AF' among patients with AF, from 2.3% in 2005 to 6.4% in 2007 and a high of 9.2% in 2013, has been reported previously.[6]

For peer review only - http://bmjopen.bmj.com/site/about/guidelines.xhtml

Since the introduction of AF into QOF, the majority of 'resolved AF' codes have been recorded between the months of January and March, immediately prior to QOF report submission by general practices. Prior to this, 'resolved AF' codes were recorded throughout the year with little monthly variation in incidence. There is no difference in stroke/TIA rates in patients diagnosed as having 'resolved AF' between January and March compared to those diagnosed later in the year; patients with AF who are diagnosed as resolved immediately prior to QOF do not have a different/lower risk of stroke/TIA.

Immediately following the introduction of AF into QOF, there was a dramatic rise in median duration between AF and 'resolved AF' diagnoses, with a further peak at the time of changes to QOF in 2012-13. At these time points, patients designated as having 'resolved AF' had been diagnosed with AF several years previously (median 3 years and 8 months in 2006) compared around one year prior to QOF (9 months in 2005).

Almost two thirds of patients with 'resolved AF' and a CHA_2DS_2-VASc score ≥1 had never been prescribed anticoagulants. In 2016, 79.5% of patients with 'resolved AF' and a CHA_2DS_2-VASc score ≥1 were not prescribed anticoagulants at the time of their 'resolved AF' diagnosis, made up of 53.5% who had never been prescribed anticoagulants and 26.0% who had previously been prescribed anticoagulants but had subsequently discontinued. By contrast, only 25-30% of patients with ongoing AF and a CHA_2DS_2-VASc score ≥1 were not prescribed anticoagulants in 2016.[8,15] This suggests that patients with AF who are not prescribed anticoagulants may be more likely to be assigned a 'resolved AF' code. Furthermore, recent evidence indicates that patients with a diagnosis of 'resolved AF' remain at increased risk of stroke/TIA and may therefore benefit from continued anticoagulant prophylaxis.[6] The concept of 'resolved AF' may be delusive; AF which has apparently resolved, even following ablation, may recur.[16,17,18]

Use of the 'resolved AF' code varies between practices. Some practices with large numbers of AF patients use the code for very few patients, while others assign the code to more than a quarter of AF patients.

Strengths and limitations

This analysis was performed in a large general practice dataset which is generalisable to the UK population. Data was derived from routinely clinical data which is used by general practitioners for clinical decision-making. The use and interpretation of the 'resolved AF' clinical code is likely to vary between general practitioners and practices. The primary care dataset contains no direct information on general practitioners' reasons for assigning a 'resolved AF' code; possible influencing factors have therefore been inferred from explorations of temporal variation, patient diagnostic information and anticoagulant prescribing. In order to better understand the factors motivating a diagnosis of 'resolved AF', a qualitative study and consultation with practicing clinicians would be required.

Anticoagulation rates may be underestimated if treatment is managed entirely in secondary care; however, the majority of anticoagulants are prescribed in primary care. AF clinical guidelines and stroke risk scoring systems have changed over the study period; for the purpose of this study, we used current guidance (eligibility for anticoagulation based on CHA_2DS_2-VASc score ≥1) across all time periods for consistency and comparability.

For peer review only - http://bmjopen.bmj.com/site/about/guidelines.xhtml

Conclusions

Use of the ‘resolved AF’ code remains common. Most patients eligible for anticoagulant treatment who were assigned a ‘resolved AF’ code were never prescribed anticoagulants, and very few patients were still taking anticoagulants when the ‘resolved AF’ code was recorded. Those diagnosed as having ‘resolved AF’ are no longer included in the AF register for QOF; this has the effect of improving the practice’s apparent performance in the QOF. Incidence of the ‘resolved AF’ clinical code increased markedly when AF was introduced into QOF in 2006 and increased again when further changes were made to the QOF incentive scheme in 2012. Since 2006, incidence of the ‘resolved AF’ code has been highest in the months shortly before practices make their QOF submissions. Previous evidence demonstrated patients with a diagnosis of ‘resolved AF’ remain at increased risk of stroke/TIA and are therefore likely to benefit from anticoagulant prophylaxis. We therefore recommend that patients with ‘resolved AF’ should be included when determining whether practices meet QOF clinical performance targets.

For peer review only

For peer review only - http://bmjopen.bmj.com/site/about/guidelines.xhtml

FIGURE LEGENDS

Figure 1. Annual incidence of resolved atrial fibrillation in patients with AF 2000-2016.

Figure 2. Incidence of the 'resolved AF' code by month of recording, before, during and after the introduction of AF into the Quality and Outcomes Framework (QOF).

Figure 3. Funnel plot showing variation in use of the 'resolved AF' code by practice in 2016.

ETHICAL APPROVAL

The THIN data collection scheme and research carried out using THIN data were approved by the NHS South-East Multicentre Research Ethics Committee (MREC) in 2003; under the terms of this approval, studies must undergo independent scientific review. Approval for these analyses was obtained from the Scientific Review Committee (for the use of THIN data) in April 2015 (15THIN021) and September 2017 (SRC reference number 17THIN082).

COMPETING INTERESTS

All authors have completed the ICMJE uniform disclosure form at http://www.icmje.org/coi_disclosure.pdf and declare: NA and TM report a grant from the National Institute for Health Research (NIHR) Collaboration for Leadership in Applied Health Research and Care West Midlands during the conduct of the study; KN reports funding from AstraZeneca and fees from Sanofi and Boehringer Ingelheim outside the submitted work. Authors declare no other financial relationships with any organisations that might have an interest in the submitted work in the previous three years; and no other relationships or activities that could appear to have influenced the submitted work.

CONTRIBUTORS

NA, KN and TM designed the study. KN undertook data extraction. NA designed and performed the analyses. NA wrote the first draft of the paper, which was revised in collaboration with TM and KN. NA acts as guarantor.

FUNDING

NJA and TM were funded by the NIHR Collaboration for Leadership in Applied Health Research and Care West Midlands initiative (NIHR CLAHRC-WM). This paper presents independent research and the views expressed are those of the author(s) and not necessarily those of the NHS, the NIHR or the Department of Health.

The funders had no role in study design, data collection and analysis, decision to publish, or preparation of the manuscript.

For peer review only

For peer review only - http://bmjopen.bmj.com/site/about/guidelines.xhtml

TRANSPARENCY

The lead author (the manuscript's guarantor) affirms that the manuscript is an honest, accurate, and transparent account of the study being reported; that no important aspects of the study have been omitted; and that any discrepancies from the study as planned (and, if relevant, registered) have been explained.

DATA SHARING STATEMENT

Dataset is not available.

EXCLUSIVE LICENCE

I, the Submitting Author has the right to grant and does grant on behalf of all authors of the Work (as defined in the below author licence), an exclusive licence and/or a non-exclusive licence for contributions from authors who are: i) UK Crown employees; ii) where BMJ has agreed a CC-BY licence shall apply, and/or iii) in accordance with the terms applicable for US Federal Government officers or employees acting as part of their official duties; on a worldwide, perpetual, irrevocable, royalty-free basis to BMJ Publishing Group Ltd ("BMJ") its licensees and where the relevant Journal is co-owned by BMJ to the co-owners of the Journal, to publish the Work in BMJ Open and any other BMJ products and to exploit all rights, as set out in our licence.

For peer review only

REFERENCES

[1] Antithrombotic Trialists' Collaboration. Collaborative meta-analysis of randomised trials of antiplatelet therapy for prevention of death, myocardial infarction, and stroke in high risk patients. *BMJ* 2002; 324:71–86.

[2] Aguilar MI and Hart R. Oral anticoagulants for preventing stroke in patients with non-valvular atrial fibrillation and no previous history of stroke or transient ischemic attacks. Cochrane Database of Systematic Reviews 2005, Issue 3. Art. No.: CD001927. doi: 10.1002/14651858.CD001927.pub2.

[3] Aguilar MI, Hart R and Pearce LA. Oral anticoagulants versus antiplatelet therapy for preventing stroke in patients with non-valvular atrial fibrillation and no history of stroke or transient ischemic attacks. *Cochrane Database of Systematic Reviews* 2007, Issue 3. Art. No.: CD006186. doi: 10.1002/14651858.CD006186.pub2.

[4] Wynn GJ, El-Kadri M, Haq I, Das M, Modi S, Snowdon R, Hall M, Waktare JE, Todd DM, Gupta D. Long-term outcomes after ablation of persistent atrial fibrillation: an observational study over 6 years. Open Heart. 2016;3:e000394. doi: 10.1136/openhrt-2015-000394

[5] Militaru C, Donoiu I. Atrial Fibrillation Recurrence Predictors after Conversion to Sinus Rhythm Curr Health Sci J. 2014 Apr-Jun; 40(2): 129–133. doi:10.12865/CHSJ.40.02.09

[6] Adderley NJ, NIrantharakumar K, Marshall T. Risk of stroke and transient ischaemic attack in patients with a diagnosis of resolved atrial fibrillation: retrospective cohort studies. BMJ 2018. doi:10.1136/bmj.k1717

[7] NHS Employers and General Practitioners Committee. Revisions to the GMS contract 2006/07. Delivering investment in general practice. NHS Employers: London 2006.

[8] Adderley NJ, Ryan R, Nirantharakumar K, Marshall T. Prevalence and treatment of atrial fibrillation in UK general practice from 2000 to 2016. Heart 2018. doi:10.1136/heartjnl-2018-312977

[9] NHS Employers and General Practitioners Committee. Quality and Outcomes Framework for 2012/13. Guidance for PCOs and practices. NHS Employers: London 2012.

[10] Maguire A, Blak BT, Thompson M. The importance of defining periods of complete mortality reporting for research using automated data from primary care. *Pharmacoepidemiol Drug Saf* 2009;18:76e83.

[11] Cowan C, Healicon R, Robson I, et al. The use of anticoagulants in the management of atrial fibrillation among general practices in England. *Heart* 2013;99:1166–72. doi: 10.1136/heartjnl-2012-303472

[12] Adderley N, Ryan R, Marshall T. The role of contraindications in prescribing anticoagulants to patients with atrial fibrillation: a cross-sectional analysis of primary care data in the UK. *Br J Gen Prac* 2017. doi: 10.3399/bjgp17X691685

[13] Isaew A, Adderley NJ, Ryan R, Fitzmaurice D, Marshall T. The treatment of paroxysmal atrial fibrillation in UK primary care. *Heart* 2017;103:1502-7. doi: 10.1136/heartjnl-2016-310927

[14] NHS England. New GMS Contract QOF Implementation. Dataset and Business Rules. Atrial Fibrillation Indicator Set. Leeds: Health and Social Care Information Centre 2016.

[15] Lacoin L, Lumley M, Ridha E, Pereira M, McDonald L, Ramagopalan S, et al. Evolving landscape of stroke prevention in atrial fibrillation within the UK between 2012 and 2016: a cross-sectional analysis study using CPRD. BMJ Open 2017;7:e015363. doi:10.1136/bmjopen-2016-015363

[16] Chao TF, Lin YJ, Chang SL, et al. Can oral anticoagulants be stopped safely after a successful atrial fibrillation ablation? *J Thorac Dis* 2015;7:172-7. doi: 10.3978/j.issn.2072-1439.2015.01.18

For peer review only - http://bmjopen.bmj.com/site/about/guidelines.xhtml

[17] Chao TF, Tsao HM, Lin YJ, et al. Clinical outcome of catheter ablation in patients with nonparoxysmal atrial fibrillation: results of 3-year follow-up. *Circ Arrhythm Electrophysiol* 2012;5:514-20.

[18] Tilz RR, Rillig A, Thum AM, et al. Catheter ablation of long-standing persistent atrial fibrillation: 5-year outcomes of the Hamburg Sequential Ablation Strategy. *J Am Coll Cardiol* 2012;60:1921-9.

For peer review only

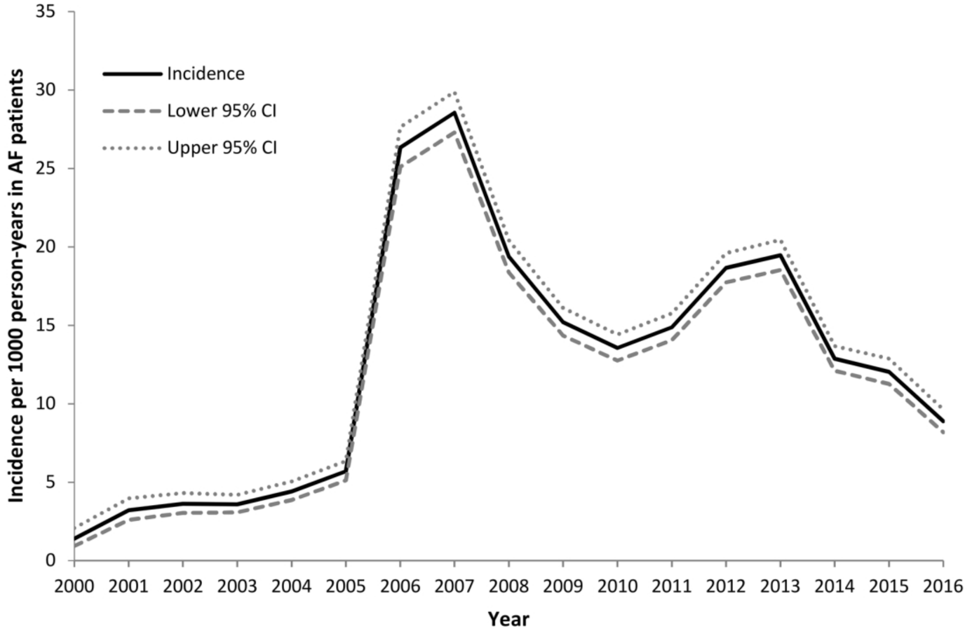

Figure 1. Annual incidence of resolved atrial fibrillation in patients with AF 2000-2016.

157x105mm (300 x 300 DPI)

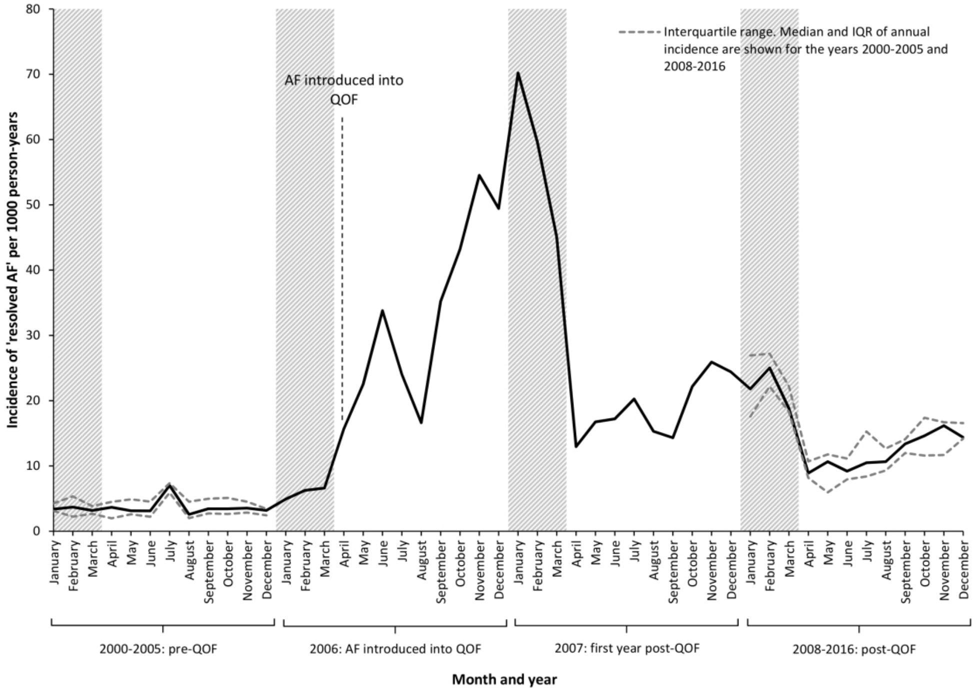

Figure 2. Incidence of the 'resolved AF' code by month of recording, before, during and after the introduction of AF into the Quality and Outcomes Framework (QOF).

223x157mm (299 x 299 DPI)

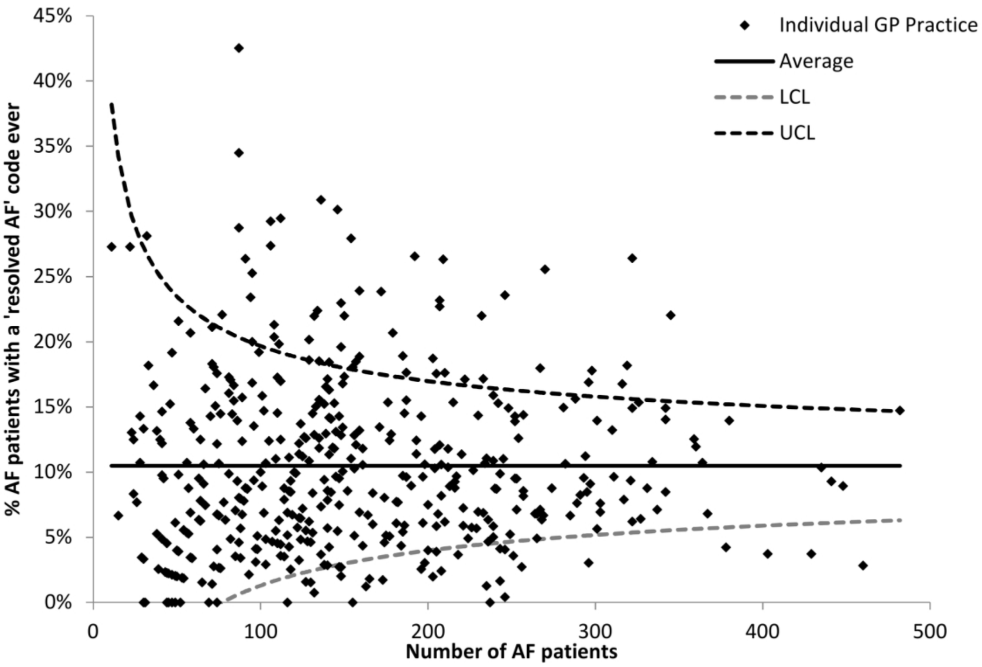

Figure 3. Funnel plot showing variation in use of the 'resolved AF' code by practice in 2016.

155x104mm (300 x 300 DPI)

The RECORD statement – checklist of items, extended from the STROBE statement, that should be reported in observational studies using routinely collected health data.

	Item No.	STROBE items	Location in manuscript where items are reported	RECORD items	Location in manuscript where items are reported
**Title and abstract**					
	1	(a) Indicate the study's design with a commonly used term in the title or the abstract (b) Provide in the abstract an informative and balanced summary of what was done and what was found	p.3	RECORD 1.1: The type of data used should be specified in the title or abstract. When possible, the name of the databases used should be included.<br><br>RECORD 1.2: If applicable, the geographic region and timeframe within which the study took place should be reported in the title or abstract.<br><br>RECORD 1.3: If linkage between databases was conducted for the study, this should be clearly stated in the title or abstract.	p.3<br><br>p.3<br><br>n/a
**Introduction**					
Background rationale	2	Explain the scientific background and rationale for the investigation being reported	p.4		
Objectives	3	State specific objectives, including any prespecified hypotheses	p.4		
**Methods**					
Study Design	4	Present key elements of study design early in the paper	pp.2, 4-6		
Setting	5	Describe the setting, locations, and relevant dates, including periods of recruitment, exposure, follow-up, and data collection	pp.4-5		

For peer review only

Participants	6	*(a) Cohort study* - Give the eligibility criteria, and the sources and methods of selection of participants. Describe methods of follow-up<br>*Case-control study* - Give the eligibility criteria, and the sources and methods of case ascertainment and control selection. Give the rationale for the choice of cases and controls<br>*Cross-sectional study* - Give the eligibility criteria, and the sources and methods of selection of participants<br><br>*(b) Cohort study* - For matched studies, give matching criteria and number of exposed and unexposed<br>*Case-control study* - For matched studies, give matching criteria and the number of controls per case	pp.5-6<br>n/a<br>pp.5-6<br>n/a<br>n/a	RECORD 6.1: The methods of study population selection (such as codes or algorithms used to identify subjects) should be listed in detail. If this is not possible, an explanation should be provided.<br><br>RECORD 6.2: Any validation studies of the codes or algorithms used to select the population should be referenced. If validation was conducted for this study and not published elsewhere, detailed methods and results should be provided.<br><br>RECORD 6.3: If the study involved linkage of databases, consider use of a flow diagram or other graphical display to demonstrate the data linkage process, including the number of individuals with linked data at each stage.	pp.5-6<br>p.5<br>n/a
Variables	7	Clearly define all outcomes, exposures, predictors, potential confounders, and effect modifiers. Give diagnostic criteria, if applicable.	pp.5-6	RECORD 7.1: A complete list of codes and algorithms used to classify exposures, outcomes, confounders, and effect modifiers should be provided. If these cannot be reported, an explanation should be provided.	pp.5-6
Data sources/ measurement	8	For each variable of interest, give sources of data and details of methods of assessment (measurement).<br>Describe comparability of assessment methods if there is more than one group	pp.5-6		

For peer review only

Bias	9	Describe any efforts to address potential sources of bias	pp.9-10		
Study size	10	Explain how the study size was arrived at	n/a – full sample		
Quantitative variables	11	Explain how quantitative variables were handled in the analyses. If applicable, describe which groupings were chosen, and why	pp.5-6		
Statistical methods	12	(a) Describe all statistical methods, including those used to control for confounding<br>(b) Describe any methods used to examine subgroups and interactions<br>(c) Explain how missing data were addressed<br>(d) *Cohort study* - If applicable, explain how loss to follow-up was addressed<br>*Case-control study* - If applicable, explain how matching of cases and controls was addressed<br>*Cross-sectional study* - If applicable, describe analytical methods taking account of sampling strategy<br>(e) Describe any sensitivity analyses	pp.5-6<br>n/a<br>n/a<br>n/a<br>n/a<br>n/a<br>n/a		
Data access and cleaning methods		..		RECORD 12.1: Authors should describe the extent to which the investigators had access to the database population used to create the study population.	p.4

For peer review only

				RECORD 12.2: Authors should provide information on the data cleaning methods used in the study.	p.5
Linkage		..		RECORD 12.3: State whether the study included person-level, institutional-level, or other data linkage across two or more databases. The methods of linkage and methods of linkage quality evaluation should be provided.	n/a
**Results**					
Participants	13	(a) Report the numbers of individuals at each stage of the study (*e.g.*, numbers potentially eligible, examined for eligibility, confirmed eligible, included in the study, completing follow-up, and analysed)<br>(b) Give reasons for non-participation at each stage.<br>(c) Consider use of a flow diagram	pp.7-8<br>n/a<br>n/a	RECORD 13.1: Describe in detail the selection of the persons included in the study (*i.e.,* study population selection) including filtering based on data quality, data availability and linkage. The selection of included persons can be described in the text and/or by means of the study flow diagram.	pp.5-7
Descriptive data	14	(a) Give characteristics of study participants (*e.g.*, demographic, clinical, social) and information on exposures and potential confounders<br>(b) Indicate the number of participants with missing data for each variable of interest<br>(c) *Cohort study* - summarise follow-up time (*e.g.*, average and total amount)	pp.7-8<br>n/a<br>pp.7-8		
Outcome data	15	*Cohort study* - Report numbers of outcome events or summary measures over time	p.7		

For peer review only

		Case-control study - Report numbers in each exposure category, or summary measures of exposure<br>*Cross-sectional study* - Report numbers of outcome events or summary measures	n/a<br><br><br><br>p.8		
Main results	16	(a) Give unadjusted estimates and, if applicable, confounder-adjusted estimates and their precision (e.g., 95% confidence interval). Make clear which confounders were adjusted for and why they were included<br>(b) Report category boundaries when continuous variables were categorized<br>(c) If relevant, consider translating estimates of relative risk into absolute risk for a meaningful time period	pp.7-8<br><br><br><br><br><br><br>n/a<br><br><br>n/a		
Other analyses	17	Report other analyses done—e.g., analyses of subgroups and interactions, and sensitivity analyses	n/a		
**Discussion**					
Key results	18	Summarise key results with reference to study objectives	p.9		
Limitations	19	Discuss limitations of the study, taking into account sources of potential bias or imprecision. Discuss both direction and magnitude of any potential bias	pp.9-10	RECORD 19.1: Discuss the implications of using data that were not created or collected to answer the specific research question(s). Include discussion of misclassification bias, unmeasured confounding, missing data, and changing eligibility over time, as they pertain to the study being reported.	pp.9-10

For peer review only

Interpretation	20	Give a cautious overall interpretation of results considering objectives, limitations, multiplicity of analyses, results from similar studies, and other relevant evidence	p.10		
Generalisability	21	Discuss the generalisability (external validity) of the study results	p.9		
**Other Information**					
Funding	22	Give the source of funding and the role of the funders for the present study and, if applicable, for the original study on which the present article is based	p.11		
Accessibility of protocol, raw data, and programming code		..		RECORD 22.1: Authors should provide information on how to access any supplemental information such as the study protocol, raw data, or programming code.	n/a

*Reference: Benchimol EI, Smeeth L, Guttmann A, Harron K, Moher D, Petersen I, Sørensen HT, von Elm E, Langan SM, the RECORD Working Committee. The REporting of studies Conducted using Observational Routinely-collected health Data (RECORD) Statement. *PLoS Medicine* 2015; in press.

*Checklist is protected under Creative Commons Attribution (CC BY) license.

For peer review only
